# Stomatal Decoupling From Photosynthesis Under High Temperatures Is Consistent With Stomatal Optimisation

**DOI:** 10.1111/gcb.70972

**Published:** 2026-07-02

**Authors:** Simon R. G. Jones, Georg Wohlfahrt, Andrew D. Friend, Peter J. Franks, Alexander W. Cheesman, Lucas A. Cernusak, Haoyu Diao, Xiaolong Feng, Josef Urban, Tyeen Taylor, Martijn Slot, Lina M. Mercado, Peter M. Cox

**Affiliations:** ^1^ Department of Mathematics and Statistics, Faculty of Environment, Science and Economy University of Exeter Exeter UK; ^2^ Universität Innsbruck Institut für Ökologie Innsbruck Austria; ^3^ Department of Geography and Conservation Research Institute University of Cambridge Cambridge UK; ^4^ School of Life and Environmental Sciences The University of Sydney Sydney New South Wales Australia; ^5^ College of Science and Engineering James Cook University Cairns Queensland Australia; ^6^ Research Unit of Forest and Soil Ecology Swiss Federal Institute for Forest, Snow and Landscape Research WSL Birmensdorf Switzerland; ^7^ School of Biological Sciences The University of Western Australia Perth Western Australia Australia; ^8^ Department of Forest Botany, Faculty of Forestry and Wood Technology Mendel University in Brno Brno Czechia; ^9^ Faculty of Environment, Science and Economy University of Exeter Exeter UK; ^10^ The Operator's Manual for the Planet (The OMP LLC) Gustavo Alaska USA; ^11^ Smithsonian Tropical Research Institute Balboa Panama; ^12^ UK Centre for Ecology and Hydrology Wallingford UK

**Keywords:** climate extremes, evaporative cooling, heat wave, photosynthesis, stomatal conductance, stomatal optimisation theory, thermoregulation

## Abstract

Stomatal pores on plant leaves regulate the gain of carbon through photosynthesis and the loss of water through transpiration. Through their responses to environmental conditions, stomata can constrain plant productivity and transpiration fluxes, exerting a strong control on climate feedbacks over land. Although mechanistic modelling of stomata remains a challenge, semi‐empirical and optimisation models have been successfully applied to improve the simulation of land‐atmosphere fluxes of water and carbon. Optimisation approaches assume that some aspect of plant function, such as photosynthesis or growth rate, is optimised with respect to an environmental constraint, such as available soil water. Both optimisation models and semi‐empirical models predict that stomatal conductance will increase in concert with rising photosynthetic rates as temperatures approach a thermal optimum, beyond which declines in both photosynthesis and stomatal conductance are expected. However, a growing number of experiments have found that while photosynthesis declines beyond its thermal optimum, stomatal conductance often continues to increase at high temperatures. Early modelling work suggests that this phenomenon can be captured and explained by an optimal thermoregulation strategy via increased evaporative cooling at the leaf surface. However, many stomatal conductance models that are embedded within climate and Earth System Models do not correctly account for this feedback and so cannot capture observed decoupling. Here, we demonstrate that if leaf temperature is calculated iteratively outside the optimisation scheme, as is commonly done in Earth System Models, stomatal decoupling will not be captured. However, by calculating leaf temperature in parallel with optimal stomatal conductance, we find that we are able to capture observed decoupling and improve predicted leaf temperature and gas exchange. Correctly implementing the leaf energy balance equation within stomatal optimisation models will be essential for capturing high temperature responses of forests across the globe.

## Graphical abstract

Many leaf gas exchange models predict that photosynthesis and stomatal conductance are tightly coupled and respond in parallel to changing environmental conditions. However, experiments show that when temperatures increase beyond the thermal optimum of photosynthesis, stomatal conductance may continue to rise while photosynthesis declines, facilitating an increase in evaporative cooling at the leaf level. Here we show that this ‘stomatal de‐coupling’ phenomenon can be captured by optimisation approaches, but only if the leaf energy balance is fully integrated into the optimisation scheme. Traditional methods often used in Earth System models on the other hand, which include the assumption that leaf temperature is equal to air temperature, and the iterative solution of the leaf energy balance outside the stomatal optimisation scheme, do not capture this phenomenon. In this study, we explored the impact of the evaporative cooling feedback on predictions of leaf gas exchange under different environmental conditions. We found that when the leaf energy balance was accounted for within the optimisation scheme, we were able to more accurately capture observed stomatal conductance at high temperatures. It is therefore critically important that global models include a fully integrated leaf energy balance in order to capture high temperature responses of terrestrial ecosystems in the future.

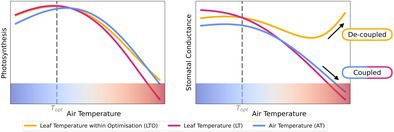

## 1 Introduction

Stomata are one of the key mediators of plant responses to climate extremes (Liang et al. 2023; Brodribb et al. 2020). Through the regulation of gas exchange at the leaf level, they play a crucial role in determining the carbon, water and energy fluxes between the land and atmosphere, and their behaviour can impact local weather and climate patterns from daily to decadal timescales (Betts et al. 2004; Hetherington and Woodward 2003; Pitman 2003; Samuel et al. 2025; Gedney et al. 2006). As a result of human‐induced climate change, many terrestrial ecosystems are facing greater intensity, duration, and frequency of both heat waves (Perkins‐Kirkpatrick and Lewis 2020) and droughts (Gebrechorkos et al. 2025), with further increases projected in the future (Domeisen et al. 2023; Cook et al. 2020). Predicting how plants will respond to these events, in particular to changes in soil moisture and increases in temperature and vapour pressure deficit (VPD) is vital for accurate projections of the global climate in the future. At present, however, these projections are uncertain. This is due largely to disagreement on the future functioning of terrestrial ecosystems (Friedlingstein et al. 2014), with projected responses to hot and dry extremes forming a key part of this model uncertainty (Sitch et al. 2008; Martínez‐de la Torre et al. 2019; De Kauwe et al. 2017; Powell et al. 2013; Paschalis et al. 2020).

Modelling stomatal behaviour is challenging. Complex signalling processes that regulate their aperture in response to environmental drivers are not well understood (Buckley et al. 2017) and species‐specific differences, for example along the iso/anisohydric continuum (Hochberg et al. 2018), complicate model development. At a global level, models are limited by the need for both computational efficiency and sufficient data to constrain parameters; hence, stomata have traditionally been modelled using empirical relationships (Jarvis 1976; Ball et al. 1987; Leuning 1995). These approaches do not explicitly represent the mechanistic processes behind stomatal behaviour but instead use fitted responses of stomata to their environmental drivers. As a result, it is unclear how robust they are to significant changes in climate, which may take plants outside their current natural environmental ranges and, importantly, outside the environmental ranges at which the empirical relationships were developed (Bonan et al. 2014). Given the lack of mechanistic underpinning within these models, there is no guarantee that empirical approaches will accurately capture the response of stomata to the climate extremes that are predicted under most climate change scenarios in the future.

In recent years stomatal optimisation theory (SOT) has emerged as a promising intermediate step between the empirical and more mechanistic approaches (Medlyn et al. 2011). At its core, SOT works on the principle that plants must open their stomata in order to facilitate the uptake of carbon dioxide (CO_2_) required for photosynthesis (and ultimately growth and reproduction), but in doing so also increase the rate of water loss through transpiration. Plants will generally try to avoid high rates of transpiration since, amongst other things, excessive water loss can lead to reduced growth via a loss of turgor pressure (Hsiao 1973; Cosgrove 2014; Fricke 2017) and an increased risk of xylem cavitation (Tyree and Sperry 1989; Martínez‐Vilalta et al. 2014; Sperry and Love 2015), which can be a precursor to hydraulic failure and ultimately plant mortality (Rowland et al. 2015). This resulting trade‐off between carbon acquisition and water retention was first exploited in the seminal work of Cowan and Farquhar (1977), who found the solution to the resulting optimisation problem produced realistic predictions of stomatal conductance with a comparatively small number of parameters. Since then numerous optimisation models have been proposed and tested (for reviews see Wang et al. 2020; Sabot et al. 2022). These models are all fundamentally built on the carbon‐water trade‐off and differ mostly in their representation of the water cost function (Wang et al. 2020). This is due in part to the wide range of roles that water availability plays in plant function, but also due to the differing representations of plant hydraulic architecture present in the global land surface models for which optimality models are often developed (Paschalis et al. 2024; Kennedy et al. 2019; Eller et al. 2020).

A key outcome of the carbon‐water trade‐off at the heart of stomatal optimisation theory is the prediction that stomatal conductance and the rate of photosynthesis are tightly coupled. When conditions are favourable for photosynthesis, stomata open to increase carbon uptake; while stomata close to avoid wasting water when photosynthesis is limited by environmental effects on non‐stomatal processes. As a result, stomatal conductance and the rate of photosynthesis tend to vary in parallel. Indeed, the observed responses of stomatal conductance to changing climatic variables, such as light, water availability, and temperature, typically mirror those of photosynthesis (Wong et al. 1979). This is evident in the generally conservative nature of the ratio of intracellular to atmospheric CO_2_ concentration ($C_{i}:C_{a}$) and reveals the critical nature of water to plant function. However, growing evidence suggests that this tight coupling can be broken under high temperatures. Specifically, it has been observed that while photosynthesis decreases with increasing temperature beyond its thermal optimum, stomatal conductance can continue to rise (Raschke 1970; De Kauwe et al. 2019; Krich et al. 2022; Marchin et al. 2023; Feng et al. 2023; Diao et al. 2024; Pankasem et al. 2024; Sadok et al. 2021; Gauthey et al. 2024). This seemingly undesirable behaviour can result in large losses of water if atmospheric vapour pressure deficit (VPD) is high, despite significant reductions in photosynthetic uptake, which contradicts the optimality principle upon which many models rely. As a result, contemporary models, in particular those that rely on a conservative $C_{i}:C_{a}$ ratio, fail to capture this behaviour. Owing to the growing prevalence of heat extremes, this likely presents a significant problem for accurate predictions of plant responses to climate change in the future. Given the importance of water, carbon and energy fluxes between the land and atmosphere (Betts et al. 2004), significant uncertainty may result from this missing process in predictions of the future climate. It is therefore important to understand this phenomenon and improve representations of stomatal behaviour, in particular within coupled Earth System Models that allow the wider impact of stomatal decoupling to be examined.

It has been hypothesised that the decoupling of photosynthesis and stomatal conductance at high temperatures represents an evolved strategy that allows plants to regulate leaf temperature through evaporative cooling (Drake et al. 2018). By increasing transpiration at high temperatures, plants can reduce leaf temperature, thereby producing more favourable conditions for photosynthesis and avoiding heat‐induced damage to photosynthetic machinery. It may therefore be beneficial overall for plants to open their stomata at high temperatures despite the fact that doing so may lead to catastrophic losses of water under certain conditions. Indeed, early work by Friend (1995) showed that this decoupling behaviour emerges from an optimisation model when the impact of stomatal conductance on leaf temperature is accounted for. However, until recently, the theory behind this feedback has gone largely unexplored (Sicangco et al. 2026). In addition, the stomatal models that are embedded within modern Earth System Models do not appear to capture this behaviour and so are at odds with these observations (Wang et al. 2026). As a result, we need to better understand the circumstances under which stomatal decoupling can be captured using optimisation approaches, and in doing so may also be able to shed light on some of the environmental conditions under which plants prioritise regulating leaf temperature over avoiding potentially fatal levels of hydraulic failure. For example, while it might be expected that a sufficient water supply would be needed for plants to employ this thermoregulation strategy, contrasting dependences of stomatal decoupling on soil moisture have been found across different studies (Marchin et al. 2022, 2023; Drake et al. 2018; Krich et al. 2022; Urban et al. 2017). This suggests a complex interaction of the phenomenon with drought and greater exploration of the theory behind stomatal decoupling within optimisation models is required. This may help to generate new hypotheses and further advance our understanding of this phenomenon in the real world.

**FIGURE 1:**
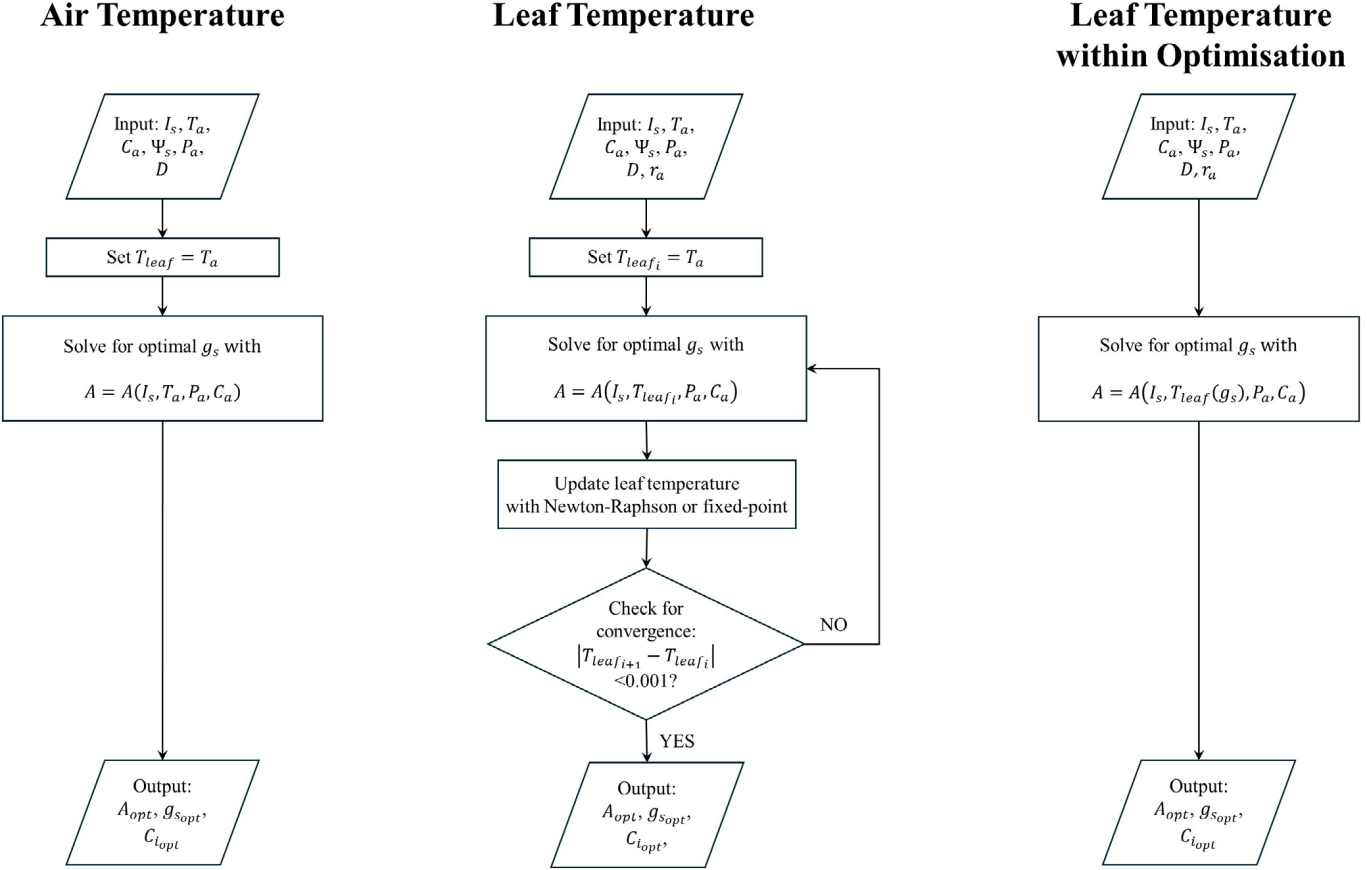
Schematic diagrams of the three approaches to the leaf energy balance within stomatal optimisation. All three methods require inputs of incoming short‐wave radiation ($I_{s}$); air temperature ($T_{a}$); atmospheric CO_2_ ($C_{a}$); soil water potential ($\Psi_{s}$); atmospheric air pressure ($P_{a}$); and vapour pressure deficit (D). In addition, the ‘Leaf Temperature’ and ‘Leaf Temperature within Optimisation’ approaches require atmospheric aerodynamic resistance to water vapour ($r_{a}$). In the ‘Air Temperature’ method leaf temperature is set equal to air temperature and optimal gas exchange is calculated. In the ‘Leaf Temperature’ approach leaf temperature is initialised as equal to air temperature and optimal gas exchange is calculated. The leaf temperature is then updated using the optimal conductance and this process is iterated until the energy balance and optimisation are consistent. In the ‘Leaf Temperature within Optimisation’ approach optimal gas exchange is calculated with leaf temperature as a function of stomatal conductance. Hence, optimal leaf temperature and stomatal conductance are calculated in parallel.

To account for the effect of evaporative cooling within a stomatal optimisation model it is necessary to represent the leaf energy balance. Most applications of stomatal optimisation theory have taken one of two approaches to the leaf energy budget. The first, which we herein refer to as the ‘Air Temperature’ (AT) approach, is to assume that leaves are perfectly thermally coupled to the atmosphere, setting leaf temperature equal to air temperature. This method, by definition, does not account for the effect of evaporative cooling and so should not be able to capture the decoupling of stomatal conductance and photosynthesis. The second approach, which we herein refer to as the ‘Leaf Temperature’ (LT) approach, is to calculate leaf temperature with an iterative method, whereby optimal stomatal conductance and leaf temperature are calculated sequentially over a repeating loop. Typically, leaf temperature is initialised with air temperature and used to calculate optimal stomatal conductance. This is in turn used to calculate leaf temperature, and the process is repeated until convergence. This type of fixed point iteration is illustrated well in figure 1 of Kim and Lieth (2003), which we recreate here (Figure 1). This approach allows a distinction between leaf and air temperature to be made, and intuition would suggest that it should facilitate stomatal decoupling. However, each time optimal stomatal conductance is calculated, it is done so with a fixed leaf temperature. While the final result is a leaf temperature and stomatal conductance that satisfies the leaf energy balance equation, this approach does not account for the benefit of opening stomata at high temperatures within the optimisation scheme. As we demonstrate within this work, this does not allow stomatal decoupling to be captured.

To our knowledge, Friend (1995) was the first to explicitly integrate the leaf energy balance equation within an optimisation scheme such that leaf temperature and optimal stomatal conductance are calculated in parallel. This method which we refer to as the ‘Leaf Temperature within Optimisation’ (LTO) method allows leaf temperature to vary during the calculation of optimal conductance and as a result facilitates the decoupling of stomatal conductance and photosynthesis. Despite this early work, few studies appear to have employed this approach and those that have, have not focused on the phenomenon of stomatal decoupling (Sabot et al. 2020). More recently, however, Sicangco et al. (2026) were the first to implement a fully integrated leaf energy balance within an optimisation model with the explicit goal of examining the high temperature response of stomata. While they demonstrated stomatal decoupling within the model, they found limited improvement in its capability to predict observed gas exchange during heat waves at a canopy level. In this study we have two overarching goals. The first is to further explore the capacity of optimisation approaches to explain observed decoupling, and to examine some of the uncertainties surrounding the conditions under which decoupling may occur, specifically the predicted drought response. The second goal is to demonstrate the difference between the three approaches to leaf temperature described above. In particular, we look to show how the subtle differences between the ‘LTO’ and ‘LT’ approaches have an important impact on the predicted responses at high temperatures. To achieve these aims, we first explored the theoretical temperature response of a stomatal optimisation model using all three methods (LTO, LT, AT). Next we used the fully integrated model (LTO) to explore the theoretical role of soil moisture availability on the predicted decoupling phenomenon. We then tested the LTO and LT models against measured data and show that only the LTO approach is able to capture observed decoupling patterns. Finally, we explored the implications of our findings for land surface modelling, in particular how it affects the commonly used ‘Medlyn stomatal model’ (Medlyn et al. 2011).

## 2 Methods

### 2.1 Model Description

We used a simplified version of the PGENv2 model (Friend 1995) in which we implemented an instantaneous optimisation approach. The model assumes stomatal conductance to CO_2_ ($g_{\mathrm{sc}}$; mol m^−2^ s^−1^) maximises the instantaneous product of net leaf photosynthesis ($A_{n}$; mol m^−2^ s^−1^) and a unit‐less water cost function ($f_{\psi_{\text{leaf}}}$). We made a number of simplifications to the representation of carbon and water fluxes relative to the original PGENvn2 model and we describe the simplified model below. A full list of symbols used can be found in Table 1.

**TABLE 1:** A list of symbols.

Symbol	Definition	Default value	Units
$A_{n}$	Net photosynthesis		mol m^−2^ s^−1^
$A_{C}$	Carboxylation‐limited photosynthesis		mol m^−2^ s^−1^
$A_{J}$	RuBP regeneration‐limited photosynthesis		mol m^−2^ s^−1^
c	Speed of light	$3\times 10^{8}$	m s^−1^
$C_{a}$	Atmospheric CO_2_ partial pressure	40.0	Pa
$C_{i}$	Internal leaf CO_2_ partial pressure		Pa
$c_{p}$	Heat capacity of air	1003.5	J kg^−1^ K^−1^
D	Vapour pressure deficit		Pa
E	Transpiration		mol m^−2^ s^−1^
e	Vapour pressure of water		Pa
$e_{s}$	Saturated vapour pressure of water		Pa
$f_{\psi_{\text{leaf}}}$	Leaf water potential factor		Dimensionless
$g_{\text{crit}}$	Critical stomatal conductance to CO_2_ at the water cost function becomes zero		mol m^−2^ s^−1^
$g_{\mathrm{sc}}$	Stomatal conductance to CO_2_		mol m^−2^ s^−1^
h	Planck constant	$6.626\times 10^{-34}$	M^2^ kg s^−1^
$E_{a}$	Energy of activation for photosynthesis	$50\times 10^{3}$	J mol^−1^
$H_{d}$	Energy of deactivation for photosynthesis	$200\times 10^{3}$	J mol^−1^
$I_{\text{APAR}}$	Absorbed photosynthetically active radiation		${\mathrm{mol}}_{\text{quanta}}$ m^−2^ s^−1^
$I_{\mathrm{abs}}$	Absorbed short‐wave radiation		W m^−2^
$I_{s}$	Incident short‐wave radiation		W m^−2^
J	Potential rate of electron transport		mol m^−2^ s^−1^
$J_{\max}$	Light‐saturated electron transport rate		mol m^−2^ s^−1^
$K_{c}$	Michaelis–Menten parameter for CO_2_		Pa
$K_{o}$	Michaelis–Menten parameter for O_2_		Pa
$k_{T_{\max}}$	The maximum rates of $V_{\text{cmax}}$ and $J_{\max}$ at their thermal optima	39.5 × 10^−6^, 63.2 × 10^−6^	mol m^−2^ s^−1^
$N_{a}$	Avagadro's constant	$6.022\;\times 10^{23}$	mol^−1^
$P_{a}$	Atmospheric air pressure	101325.0	Pa
$q_{10}$	Q_10_ value for day respiration	2.0	Dimensionless
$q_{10_{\tau }}$	Q_10_ value for the specificity of Rubisco	0.57	Dimensionless
$r_{a}$	Aerodynamic resistance to water vapour	10.0	s m^−1^
$r_{\mathrm{HR}}$	Parallel resistance to heat loss by convection and radiation		s m^−1^
$r_{p}$	Total hydraulic resistance between soil and leaf	200	MPa m^2^ s mol^−1^
$r_{\mathrm{sv}}$	Stomatal resistance to water vapour		s m^−1^
R	Molar gas constant	8.314462	J K^−1^ mol^−1^
$R_{d}$	Mitochondrial day respiration		mol m^−2^ s^−1^
s	Slope of saturated vapour pressure with respect to temperature		Pa K^−1^
$T_{a}$	Atmospheric temperature		°C
$T_{b}$	Background temperature		°C
$T_{\text{leaf}}$	Leaf temperature		°C
$T_{\mathrm{opt}}$	Optimal leaf temperature	35.0	°C
$T_{s}$	Apparent radiative temperature of the atmosphere		°C
$V_{\text{cmax}}$	Maximum rate of carboxylation		mol m^−2^ s^−1^
α	Apparent quantum yeild of electron transport	0.3	${\mathrm{mol}}_{\text{electron}}$ ${\mathrm{mol}}_{\text{photon}}^{-1}$
γ	Thermodynamic psychrometric constant		mol m^−3^ K^−1^
$\Gamma^{*}$	CO_2_ compensation point in the absence of dark respiration		Pa
$\lambda_{\mathrm{PAR}}$	Average wavelength of photosynthetically active radiation	550	nm
$\lambda_{v}$	Latent heat of vapourisation of H_2_O	2.501 × 10^6^	J kg^−2^
ρ	Density of air		kg m^−3^
σ	Stefan‐Boltzman constant	5.67 × 10^8^	W m^−2^ K^−4^
τ	Specificity of Rubisco		Dimensionless
$\tau_{25}$	Specificity of Rubisco at 25°C	2710	Dimensionless
Φ	Isothermal net absorbed radiation		W m^−2^
$\Psi_{\text{crit}}$	Critical leaf water potential at which dry matter production is zero	−2.0	MPa
$\Psi_{\text{leaf}}$	Leaf water potential		MPa
$\Psi_{\mathrm{pd}}$	Pre‐dawn leaf water potential		MPa

As in Friend (1995), the optimal value of $g_{\mathrm{sc}}$ is given by the solution of:

(1) $$\frac{\partial \left(A\cdot f_{\psi_{\text{leaf}}}\right)}{\partial g_{\mathrm{sc}}}=0$$

#### 2.1.1 Leaf Photosynthesis C3

Leaf photosynthesis of C3 plants is calculated as the minimum of two potentially limiting rates following Farquhar et al. (1980). We apply a smoothing to the minimum to ensure continuous responses of photosynthesis and stomatal conductance to environmental changes. Net photosynthesis is therefore given by the solution to the following quadratic:

(2) $$aA_{n}^{2}-\left(A_{C}+A_{J}\right)A_{n}+A_{C}A_{J}=0$$

where a=0.9 is the smoothing parameter, and $A_{C}$ and $A_{J}$ are carboxylation‐limited photosynthesis (Equation 3), and Ribulose‐bisphosphate (RuBP) regeneration‐limited photosynthesis (Equation 4), respectively.

The carboxylation‐limited rate is given by:

(3) $$A_{C}=\frac{V_{\text{cmax}}\left(C_{i}-\Gamma^{*}\right)}{C_{i}+K_{c}\left(1+O_{a}/K_{o}\right)}-R_{d}$$

where $C_{i}$ (Pa) is the internal leaf CO_2_ partial pressure; $\Gamma^{*}$ (Pa) is the CO_2_ compensation point in the absence of dark respiration; $O_{a}$ (Pa) is the atmospheric partial pressure of oxygen (O_2_); $K_{c}$ and $K_{o}$ (Pa) are Michaelis–Menten constants of Rubisco for CO_2_ and O_2_ respectively; $R_{d}$ (mol m^−2^ s^−1^) is mitochondrial day respiration; and $V_{\text{cmax}}$ (mol m^−2^ s^−1^) is the maximum rate of carboxylation.

The RuBP regeneration‐limited rate of net photosynthesis is given by:

(4) $$A_{J}=\frac{J}{4}\frac{C_{i}-\Gamma^{*}}{C_{i}+2\Gamma^{*}}-R_{d}$$

where J (mol m^−2^ s^−1^) is the rate of electron transport, calculated following Medlyn et al. (2002):

(5) $$\theta J^{2}-\left(\alpha I_{\text{APAR}}+J_{\max}\right)+\alpha I_{\text{APAR}}J_{\max}=0$$

where $I_{\text{APAR}}$ (mol_quanta_ m^−2^ s^−1^) is the absorbed photosynthetically active radiation, $J_{\max}$ (mol m^−2^ s^−1^) is the light‐saturated potential electron transport rate, α is the apparent quantum yield of electron transport (${\mathrm{mol}}_{\text{electrons}}$ ${\mathrm{mol}}_{\text{photons}}^{-1}$), and θ is a unitless smoothing parameter which takes the value of 0.9 (Medlyn et al. 2002).

$I_{\text{APAR}}$ is calculated from absorbed short‐wave radiation ($I_{\mathrm{abs}}$, W m^−2^) assuming it makes up 50% of total absorbed radiation, and has an average wavelength of 550 nm:

(6) $$I_{\text{APAR}}=\frac{0.5\lambda_{\mathrm{PAR}}}{\mathrm{hc}N_{a}}I_{\mathrm{abs}}$$

where $\lambda_{\mathrm{PAR}}=550$ nm is the wavelength of PAR; $h=6.626\times 10^{-34}$ m^2^ kg s^−1^ is the Planck constant; $c=3.0\times 10^{8}$ ms^−1^ is the speed of light; and $N_{a}=6.022\times 10^{23}$ mol^−1^ is Avagadro's constant.

Throughout this study we typically use absorbed short‐wave radiation as an input to the model however, it can also be calculated from incident short‐wave radiation ($I_{s}$) by assuming a leaf scattering coefficient (ω) of 0.15:

(7) $$I_{\mathrm{abs}}=\left(1-\omega \right)I_{s}$$

Both $V_{\text{cmax}}$ and $J_{\max}$ are assumed to depend on leaf temperature ($T_{\text{leaf}}$; °C) following the peaked Arrhenius function (Bernacchi et al. 2002) given by:

(8) $$k\left(T_{\text{leaf}}\right)=\frac{\exp\left[c-E_{a}/RT_{k}\right]}{1+\exp\left[\left(\Delta ST_{k}-H_{d}\right)/RT_{k}\right]}$$

where k is either $V_{\text{cmax}}$ or $J_{\max}$; $E_{a}$ and $H_{d}$ (Jmol^−1^) describe the rate of exponential increase and decrease, below and above the optimum respectively; and R (JK^−1^ mol^−1^) is the molar gas constant.

The CO_2_ compensation point in the absence of dark respiration ($\Gamma^{*}$) is given by:

(9) $$\Gamma^{*}=\frac{O_{a}}{2\tau }$$

where τ (unitless) is the specificity of Rubisco for CO_2_ given by:

(10) $$\tau =\tau_{25}q_{10_{\tau }}^{0.1\left(T_{\text{leaf}}-25.0\right)}$$

where $q_{10_{\tau }}=0.57$ is the rate of change of τ per 10°C of temperature change, and $\tau_{25}$ (unitless) is the Rubisco specificity at 25°C with default value of 2710 following von Caemmerer et al. (1994).

The Michaelis–Menten constants of Rubsico ($K_{c}$ and $K_{o}$) depend on leaf temperature following Sharkey et al. (2007):

(11) $$K_{c}=\exp\left(35.9774-\frac{80.99}{0.008134\left(273.15+T_{\text{leaf}}\right)}\right)$$

(12) $$K_{o}=\exp\left(12.3772-\frac{23.72}{0.008134\left(273.15+T_{\text{leaf}}\right)}\right)\times 10^{3}$$

Finally, we assume that mitochondrial day respiration ($R_{d}$) is given by a Q_10_ function of leaf temperature (Ryan 1991):

(13) $$R_{d}=r_{d_{25}}q_{10}^{0.1\left(T_{\text{leaf}}-25\right)}$$

where $r_{d_{25}}$ is the rate of respiration at 25°C, and $q_{10}$ describes the change in respiration rate per 10°C change in temperature. We assume as a default that $r_{d_{25}}$ is equal to 1% of $V_{c_{\max}}$ at 25°C, and that $q_{10}$ has a value of 2.0.

#### 2.1.2 Leaf Photosynthesis C4

Leaf photosynthesis of C4 plants is calculated as the minimum of three potentially limiting rates following the model of Collatz et al. (1992):

(14) $$A=\min\left\{A_{c},A_{l},A_{e}\right\}-R_{d}$$

where $A_{c}$ is the carboxylation‐limited rate given by:

(15) $$A_{c}=V_{c_{\max}}-R_{d}$$

Both $V_{c_{\max}}$ and $R_{d}$ are calculated as for C3 plants using Equations (8) and (13) respectively.

$A_{l}$ is a light‐limited rate given by:

(16) $$A_{l}=\alpha I_{\text{APAR}}-R_{d}$$

and $A_{e}$ is the PEPCarboxylase limited rate given by:

(17) $$A_{e}=2\times 10^{4}V_{c_{\max}}\frac{c_{i}}{P_{a}}-R_{d}$$

where $P_{a}$ is atmospheric pressure (Pa).

#### 2.1.3 The Role of Leaf Water Potential

As in Friend (1995), the cost function of water ($f_{\psi_{\text{leaf}}}$) is given by a linear function that represents the response of dry matter production to leaf water potential ($\Psi_{\text{leaf}}$; MPa):

(18) $$f_{\psi_{\text{leaf}}}=\left\{\begin{array}{ll} 1-\frac{\Psi_{\text{leaf}}}{\Psi_{\text{crit}}} & \text{for}\;\Psi_{\text{leaf}}>\Psi_{\text{crit}}\\ 0 & \text{for}\;\Psi_{\text{leaf}}<\Psi_{\text{crit}} \end{array}\right.$$

where $\Psi_{\text{crit}}$ (MPa) is a constant and represents the minimum (most negative) possible leaf water potential, below which dry matter production is zero.

Leaf water potential is calculated as follows:

(19) $$\Psi_{\text{leaf}}=\Psi_{\mathrm{pd}}-r_{p}E$$

where $\Psi_{\mathrm{pd}}$ (MPa) is predawn leaf water potential; $r_{p}$ is the total plant hydraulic resistance between soil and leaf (MPa m^2^ s mol^−1^); and E (mol m^−2^ s^−1^) is the rate of transpiration.

The dependence of transpiration on stomatal conductance is given by:

(20) $$E=\frac{1.6Dg_{\mathrm{sc}}}{P_{a}}$$

where 1.6 is the ratio of the diffusivities of water vapour and CO_2_; and D (Pa) is internal leaf to air vapour pressure deficit (VPD) assuming air saturation within the leaf.

We note here that the cost function can also be written as follows:

(21) $$f_{\psi_{\text{leaf}}}=\left\{\begin{array}{ll} \frac{\Psi_{\text{crit}}-\Psi_{\mathrm{pd}}}{\Psi_{\text{crit}}}\left(1-\frac{g_{\mathrm{sc}}}{g_{\text{crit}}}\right) & \text{for}\;g_{\mathrm{sc}}<g_{\text{crit}}\\ 0 & \text{for}\;g_{\mathrm{sc}}\geq g_{\text{crit}} \end{array}\right.$$

where $g_{\text{crit}}$ is the critical stomatal conductance at which the cost function becomes zero. It is given mathematically by:

(22) $$g_{\text{crit}}=\frac{\beta }{D}$$

where

(23) $$\beta =\frac{\Psi_{\mathrm{pd}}-\Psi_{\text{crit}}}{1.6r_{p}}$$

This is a useful way to write the cost function for cases when the soil water potential (and hence pre‐dawn water potential) is unknown but can be assumed constant. In these cases, the term outside the bracket in Equation (21) becomes a constant and does not affect the solution for optimal $g_{\mathrm{sc}}$. Assuming that plant hydraulic resistance ($r_{p}$) is also constant allows β to be fitted to the data as a parameter. In the rare case that VPD (D) is also constant then the same can be applied to $g_{\text{crit}}$. We follow this procedure of treating β as a parameter when fitting the model to observed data in Section 2.3.

#### 2.1.4 Leaf Energy Balance

Leaf temperature is calculated using isothermal radiation and the assumption of parallel heat loss described in Jones (2013):

(24) $$T_{\text{leaf}}=T_{a}+\left(\frac{\Phi \left(r_{a}+r_{\mathrm{sv}}\right)\gamma }{\rho c_{p}}-D\right){\left(\frac{\gamma \left(r_{a}+r_{\mathrm{sv}}\right)}{r_{\mathrm{HR}}}+s\right)}^{-1}$$

where Φ (W m^−2^) is isothermal net radiation absorbed by the leaf; where $r_{a}$ (s m^−1^) is aerodynamic resistance to water vapour; $r_{\mathrm{sv}}$ (s m^−1^) is the stomatal resistance to water vapour; γ (mol m^−3^ K^−1^) is the thermodynamic psychrometric constant; ρ (kg m^−3^) is the density of air; $c_{p}$ (J kg^−1^ K^−1^) is the heat capacity of air; D (Pa) is the leaf‐to‐air vapour pressure deficit; $r_{\mathrm{HR}}$ (s m^−1^) is the parallel resistance to heat loss from the leaf by convection and radiation; and s (Pa K^−1^) is the gradient of saturated vapour pressure with respect to temperature evaluated at air temperature.

Isothermal radiation is calculated following Jones (2013):

(25) $$\Phi =I_{\mathrm{abs}}+\sigma {\left(T_{b}+253.15\right)}^{4}+\sigma {\left(T_{s}+273.15\right)}^{44}-2\sigma {\left(T_{a}+273.15\right)}^{4}$$

where σ (W m^−2^ K^−4^) is the Stefan‐Boltzman constant; $T_{b}$ (°C) is the background temperature (assumed equal to air temperature), and $T_{s}$ (°C) is the apparent radiative temperature of the atmosphere which is assumed to be 20 K lower than the actual air temperature (Jones 2013):

(26) $$T_{s}=T_{a}-20$$

The stomatal resistance to water vapour is related to $g_{\mathrm{sc}}$ by:

(27) $$r_{\mathrm{sv}}=\frac{P_{a}}{1.6g_{\mathrm{sc}}R\left(T_{a}+273.15\right)}$$

The psychrometric constant is given by:

(28) $$\gamma =\frac{P_{a}c_{p}}{0.622\lambda_{v}}$$

where $\lambda_{v}$ (J kg^−1^) is the latent heat of vapourisation of H_2_O.

Parallel resistance to heat loss from the leaf by convection and radiation is calculated following Jones (2013):

(29) $$r_{\mathrm{HR}}=\frac{1}{0.8/r_{a}+4\sigma {\left(T_{a}+273.15\right)}^{3}/\left(\rho c_{p}\right)}$$

The factor of 0.8 relates the aerodynamic resistance to water vapour ($r_{a}$) to the boundary layer resistance convective heat flux, and is equivalent to the coefficients given in Friend (1995).

The slope of saturated vapour pressure is given by:

(30) $${\left.s=\frac{de_{s}}{\mathrm{dT}}\right|}_{T_{a}}$$

where $e_{s}$ (Pa) is the saturated vapour pressure of water.

#### 2.1.5 Methods for Integrating the Leaf Energy Balance Within Stomatal Optimisation Theory

Within this work we aimed to evaluate how different methods of integrating the leaf temperature (Equation 24) within the stomatal optimisation scheme impact its predictions. Here we outline three approaches to determining leaf temperature in conjunction with a stomatal optimisation model. We refer to the three approaches as the ‘Air Temperature’ (AT), ‘Leaf Temperature’ (LT), and ‘Leaf Temperature within Optimisation’ (LTO) approaches. These are described below and illustrated in Figure 1.

- AT The leaf temperature equation is completely removed from the optimisation scheme and optimal stomatal conductance is calculated with leaf temperature set equal to air temperature. Leaf photosynthesis is therefore calculated as a function of $c_{i}$ and $T_{a}$. Only $c_{i}$ depends on stomatal conductance:

(31) $$A=A\left(T_{a},c_{i}\left(g_{s}\right)\right)$$

- LT Optimal stomatal conductance is calculated using a fixed leaf temperature which is initially set to air temperature and then iterated until the energy balance equation is satisfied. Here we do this using the Newton–Raphson method, however this can also be achieved using other root finding algorithms or through fixed point iteration (i.e., in each loop calculated conductance is used to update leaf temperature, which is then used to re‐calculate optimal conductance. This process is repeated until convergence). During the calculation of optimal stomatal conductance, leaf photosynthesis is calculated as a function of $c_{i}$ and a fixed leaf temperature ($T_{\mathrm{lea}f_{i}}$). Only $c_{i}$ depends on stomatal conductance:

(32) $$A=A\left(T_{\mathrm{lea}f_{i}},c_{i}\left(g_{s}\right)\right)$$

- LTO The leaf energy balance equation is fully integrated within the optimisation scheme and leaf temperature is calculated in parallel with optimal conductance. When optimal stomatal conductance is calculated, leaf temperature is not fixed but is instead a function of stomatal conductance. During the calculation of optimal stomatal conductance, leaf photosynthesis is calculated as a function of a variable leaf temperature and $c_{i}$, both of which depend on $g_{s}$:

(33) $$A=A\left(T_{\text{leaf}}\left(g_{s}\right),c_{i}\left(g_{s}\right)\right)$$

The difference between the LT and LTO methods is not immediately obvious; however, as we show in this study, they produce different results. While both methods produce leaf temperature and stomatal conductance predictions that satisfy the energy balance equation, they are actually solutions to fundamentally different optimisation models. The underlying optimisation Equation (1) may be expanded using the chain rule to give:

(34) $$\frac{1}{A}\frac{\partial A}{\partial g_{\mathrm{sc}}}+\frac{1}{f_{\psi_{\text{leaf}}}}\frac{\partial f_{\psi_{\text{leaf}}}}{\partial g_{\mathrm{sc}}}=0$$

If we then consider the partial derivative of photosynthesis (A) with respect to conductance ($g_{\mathrm{sc}}$), we see that in the LTO method, when optimal stomatal conductance is calculated, both $T_{\text{leaf}}$ and $c_{i}$ change with respect to $g_{s}$ and so by the chain rule, this derivative is calculated as:

(35) $$\frac{\partial A_{\mathrm{LTO}}}{\partial g_{\mathrm{sc}}}=\frac{\partial A_{\mathrm{LTO}}}{\partial T_{\text{leaf}}}\frac{\partial T_{\text{leaf}}}{\partial g_{\mathrm{sc}}}+\frac{\partial A_{\mathrm{LTO}}}{\partial c_{i}}\frac{\partial c_{i}}{\partial g_{\mathrm{sc}}}$$

In the LT method, however, when optimal conductance is calculated, leaf temperature is fixed and hence does not change with respect to $g_{\mathrm{sc}}$. As a result the derivative of A with respect to $g_{\mathrm{sc}}$ is simply given by:

(36) $$\frac{\partial A_{\mathrm{LT}}}{\partial g_{\mathrm{sc}}}=\frac{\partial A_{\mathrm{LT}}}{\partial c_{i}}\frac{\partial c_{i}}{\partial g_{\mathrm{sc}}}$$

While iterating the energy balance equation in the LT method produces a leaf temperature that is energetically consistent with the predicted stomatal conductance, unlike the LTO method the underlying objective function does not account for the evaporative cooling effect. The two methods are therefore solving distinct optimisation problems and produce contrasting results. This difference is subtle but important, and as we demonstrate here, has a significant impact on model predictions.

### 2.2 Evaluating Model Behaviour

To explore the model's behaviour we simulated the temperature response of photosynthesis, stomatal conductance and internal leaf CO_2_, holding all inputs, with the exception of VPD constant. Instead, we held atmospheric vapour pressure ($e\left(T_{a}\right)$; Pa) constant to try to replicate as real world like conditions as possible. The corresponding change in VPD is shown in Figure S1. However, we also examined the temperature response under fixed VPD in Figure S2. Temperature was varied from 10°C to 50°C, while atmospheric CO_2_ partial pressure was held at 40 Pa; absorbed short‐wave radiation at 500 W m^−2^; aerodynamic resistance to water vapour at 10 s m^−1^; and soil water potential at −0.1 MPa. Vapour pressure was held at 500 Pa to allow a positive VPD across the whole temperature range.

We then examined the role of soil water potential in the predicted decoupling phenomenon. To do this we repeated the previous simulations using the LTO model but at a range of different soil water potential values. We determined numerically the critical air temperatures at which stomatal conductance decouples from photosynthesis, which we define as the lowest air temperature at which the gradients of photosynthesis and stomatal conductance with respect to temperature are opposite signs. Similarly, we determined the air temperature at which stomata close. The results of this are shown in Figure 4.

### 2.3 Comparing the Model to Observations

To evaluate the LTO model's ability to capture real examples of stomatal decoupling under high temperatures we collated gas exchange data from across published and some unpublished literature, for a total of 38 species across a range of environmental conditions. This included 14 species that display decoupling behaviour (Diao et al. 2024; Urban et al. 2017; Feng et al. 2023; Slot et al. 2016; Taylor 2026), as well as 24 that do not (Slot and Winter 2017). While a total of 42 species were measured in Slot and Winter (2017), we selected only the species for which we were able to determine photosynthetic parameters, and for which measured stomatal conductance could predict leaf temperature with reasonable accuracy ($R^{2}>0.5$) using Equation (24). The purpose of evaluating the model against data where stomatal decoupling is not present was to assess whether the model could not only predict decoupling but appropriately predict its absence when not observed. A comprehensive list of the data used are given in Table 2.

#### 2.3.1 Parameter Estimation

For each species we first fit the photosynthesis model to the observed data using non‐linear least squares. To improve numerical stability, we used the reparametrisation of the temperature response function described by Alexandrov and Yamagata (2007) in which Equation (8) is written as:

(37) $$k\left(T_{\text{leaf}}\right)=k_{\mathrm{opt}}\frac{\eta f_{A}}{\left(\eta -1\right)+f_{A}^{\eta }}$$

where $k_{\mathrm{opt}}$ is the value of $V_{\text{cmax}}$ and $J_{\max}$ at the thermal optimum, η is the ratio of $H_{d}$ to $E_{a}$:

(38) $$\eta =\frac{H_{d}}{E_{a}}$$

and $f_{A}$ is the Arrhenius function given by:

(39) $$f_{A}\left(T_{\text{leaf}}\right)=\exp\left[\frac{E_{a}\left(T_{\text{leaf}}-T_{\mathrm{opt}}\right)}{\left(T_{\text{leaf}}+273.15\right)\left(T_{\mathrm{opt}}+273.15\right)R}\right]$$

where $T_{\mathrm{opt}}$ (°C) is the optimum leaf temperature at which electron transport or carboxylation occur (i.e., the peak of the response curve), and can be written as:

(40) $$T_{\mathrm{opt}}=\frac{H_{d}}{\Delta S-R\log\left[E_{a}/\left(H_{d}-E_{a}\right)\right]}$$

We then determine values for $V_{c_{\max,\mathrm{opt}}}$, $J_{\max,\mathrm{opt}}$, $r_{d_{25}}$, and the four temperature parameters ($E_{a}$, η, $T_{\mathrm{opt}}$, $q_{10}$). In some cases low data availability and large parameter interdependency resulted in high uncertainty in our fitted parameters. To test the implications of this on our final model predictions we selected three species and used a Markov‐Chain Monte Carlo ensemble sampler (Foreman‐Mackey et al. 2013) to determine the posterior distribution of the fitted parameters. We then took a random sample of 100 realisations from the posterior distribution and calculated predicted stomatal conductance as described below. This allowed us to determine whether the range of feasible photosynthetic parameters made a significant difference to our overall results.

Where available we used either measured absorbed or measured incident short‐wave radiation ($I_{\mathrm{abs}}$, $I_{s}$) and measured boundary layer resistance to water vapour ($r_{\mathrm{sv}}$) as inputs into the energy balance equation. However, for cases where these variables were not available we determined their values by fitting Equation (24) to observed stomatal conductance to water vapour, leaf and air temperature, and vapour pressure deficit. This was done by assuming both absorbed short‐wave radiation and boundary layer resistance were constant over the measurement period and determining their values again using non‐linear least squares.

Finally we assumed that for each dataset, soil water content and plant hydraulic resistance were constant. This allowed us to treat β (Equation 22) as a constant parameter which we could then determine again using non‐linear least squares and observed stomatal conductance. This was a reasonable assumption for data collected over short timescales but may not apply to datasets that are collected over seasonal or annual periods in which soil moisture will vary and plants may be subject to hydraulic embolisms. However, leaf water potentials and xylem hydraulic resistances are not readily measured and hence are commonly not collected alongside gas exchange measurements. The results of all parameter estimations, along with uncertainty bounds for each parameter, for each species are given in Table S1.

#### 2.3.2 Model Evaluation

To evaluate the model's ability to capture the observed data, we compared predicted photosynthesis, stomatal conductance to CO_2_, internal leaf CO_2_, and the difference in leaf and air temperature ($\mathrm{dT}=T_{\text{leaf}}-T_{a}$), from both the LTO and LT versions of PGENv2 to the measured values. We calculated *R* ^2^ values for each variable across the entire measured air temperature range. In addition, to isolate the high temperature response, we also calculated *R* ^2^ values for each variable for only data above 35°C. These results are presented in Figure 5.

### 2.4 Assessing Implications for the Medlyn Stomatal Conductance Model

The Medlyn et al. (2011) stomatal conductance model represents a link between stomatal optimisation theory and more historically used empirical models (Ball et al. 1987; Leuning 1995). It is parsimonious and given in an analytical form that allows stomatal conductance to be calculated efficiently. As a result it is widely used and has been implemented and used in a number of land surface models including ACCESS ESM1.3 (Kala et al. 2015); CESM2 (Lawrence et al. 2019); and JULES (Oliver et al. 2022). The model predicts stomatal conductance as:

(41) $$g_{\mathrm{sv}}\approx g_{0}+\left(1+\frac{g_{1}}{\sqrt{D}}\right)\frac{A}{C_{a}}$$

where $g_{0}$ and $g_{1}$ are model parameters.

This expression is often used in an empirical way, where $g_{0}$ and $g_{1}$ are fitted to data. However, the novelty of the work by Medlyn et al. (2011) was to show that this equation can be derived using an optimisation approach. This bridges the gap between empirical and optimisation models, but also provides mechanistic insights into the nature of the model parameters used in empirical equations (Lin et al. 2015). In particular, Medlyn et al. (2011) show that the theoretical definition of the $g_{1}$ parameter is given by:

(42) $$g_{1}\propto \sqrt{\Gamma^{*}\lambda }$$

where λ (mol H_2_O mol^−1^ CO_2_) is a Lagrange multiplier that describes the marginal water cost of carbon gain (Cowan and Farquhar 1977), and $\Gamma^{*}$ is again the CO_2_ compensation point in the absence of dark respiration.

However, this equation is derived without accounting for a fully integrated leaf energy balance. Following the methodology outlined in Medlyn et al. (2011) and Arneth et al. (2002), we derived a new expression for the $g_{1}$ parameter where the effect of evaporative cooling has been accounted for. A full derivation of our new expression for $g_{1}$ is given in (Notes S1). We then determined the effective $g_{1}$ parameter for the theoretical simulation performed in Section 2.2 using a rearranged form of Equation (41) for the LTO model.

## 3 Results

### 3.1 The Evaluation of Stomatal Decoupling in a Stomatal Optimisation Model

**FIGURE 2:**
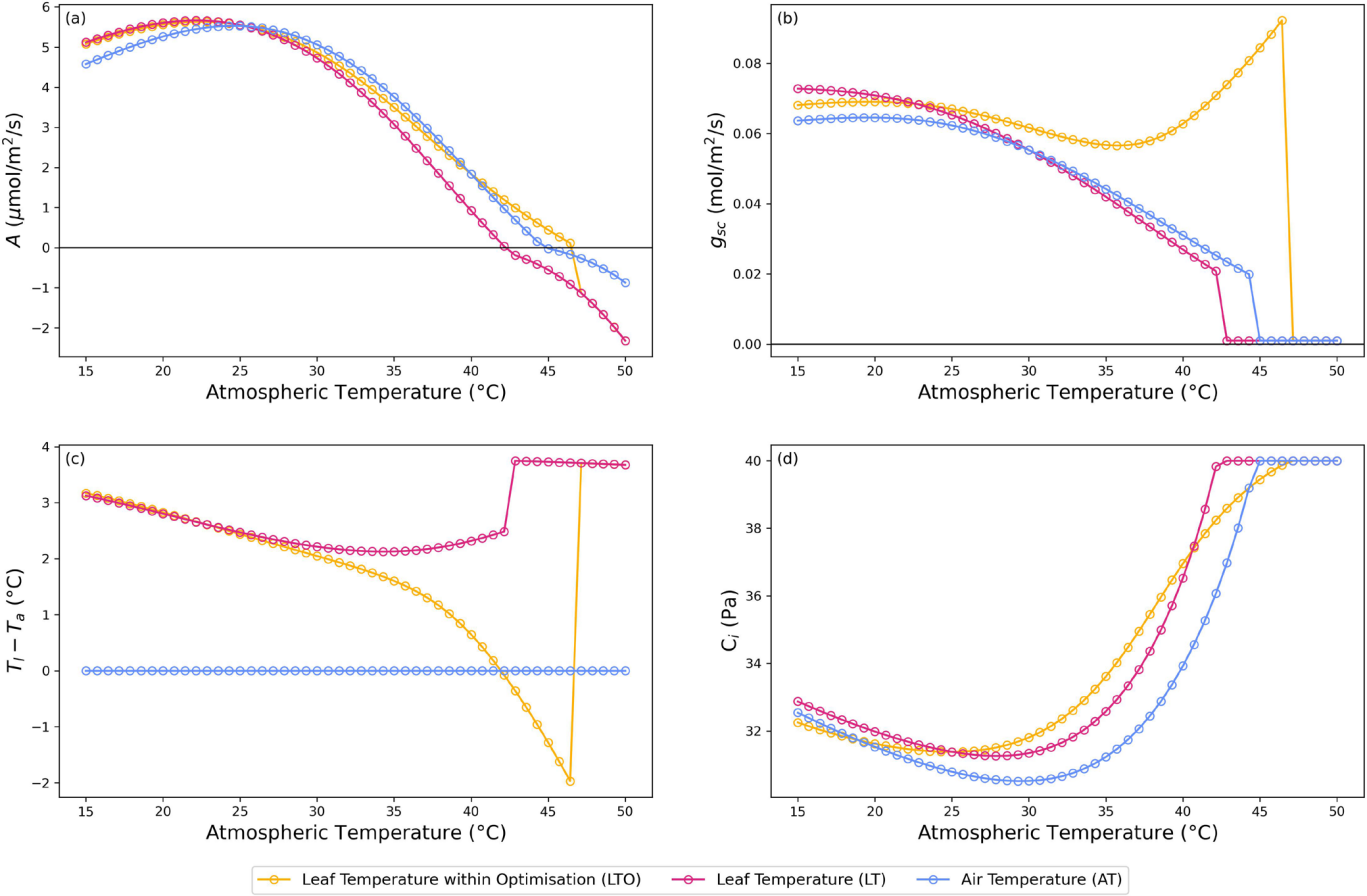
The predicted response of (a) photosynthesis, (b) stomatal conductance to CO_2_, (c) the difference between leaf and atmospheric temperature, and (d) internal leaf CO_2_ partial pressure to changes in atmospheric temperature from the simplified PGEN model. Yellow markers show the response using the ‘Leaf Temperature within Optimisation’ (LTO) approach to the leaf energy balance, pink markers show the response using the ‘Leaf Temperature’ (LT) approach, and blue markers show the response with the ‘Air Temperature’ (AT) approach. The simulations were conducted with: Atmospheric CO_2_ concentration, $C_{a}=40$ Pa; soil water potential, $\psi_{s}=-0.1$ MPa; absorbed short‐wave radiation $I_{\mathrm{abs}}=500$ W m^−2^; aerodynamic resistance to water vapour, $r_{a}=10$ s m^−1^; atmospheric vapour pressure, e=500 Pa.

**FIGURE 3:**
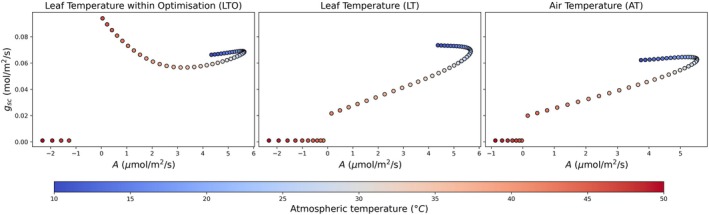
The predicted response of photosynthesis against stomatal conductance to CO_2_ from the simplified PGENvn2 model using (left) the ‘Leaf Temperature within Optimisation’ (LTO) (center) the ‘Leaf Temperature’ (LT) and (right) the ‘Air Temperature’ (AT) approaches to the leaf energy balance. Markers are coloured according to the input air temperature such that the low and high temperature responses can be distinguished. The simulations were conducted with: Atmospheric CO_2_ concentration, $C_{a}=40$ Pa; soil water potential, $\psi_{s}=-0.1$ MPa; absorbed short‐wave radiation $I_{\mathrm{abs}}=500$ W m^−2^; aerodynamic resistance to water vapour, $r_{a}=10$ s m^−1^; atmospheric vapour pressure, e=500 Pa.

When the leaf energy balance was fully accounted for in the ‘LTO’ approach, we observed decoupling behaviour at temperatures above the thermal optimum for photosynthesis (Figure 2). This behaviour was not present in either the ‘LT’ or ‘AT’ versions of the model where stomatal conductance and photosynthesis responded in parallel to changing air temperature (Figure 3). The decoupling in the ‘LTO’ approach was driven by an increased benefit of stomatal aperture due to the evaporative cooling that results from increased transpiration. This was evident from the sharp decrease in the difference between leaf and air temperature at high temperatures (Figure 2c), which was present in the ‘LTO’ simulation but not the ‘LT’ simulation or ‘AT’ simulation (the latter by definition predicted no difference between leaf and air temperature). Another important feature of the simulations was that at temperatures below the thermal optimum of photosynthesis, the ‘LTO’ and ‘LT’ approaches predict similar estimates of photosynthesis, stomatal conductance, and leaf temperature (Figure 2a–c). It is only when the decoupling occurred that the two approaches diverged significantly.

**FIGURE 4:**
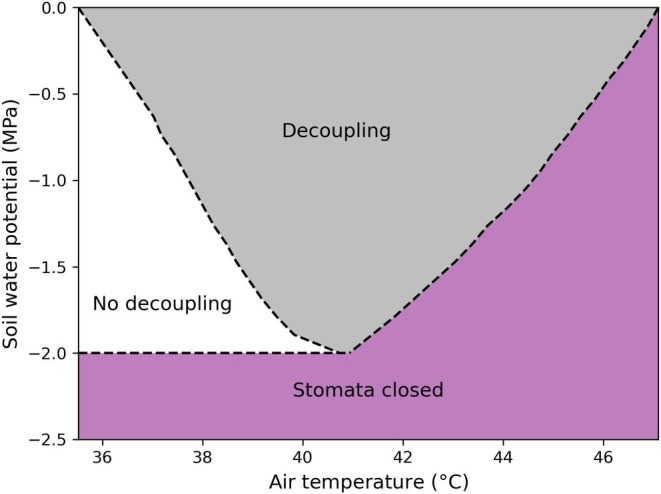
The dependence of decoupling on soil water potential and air temperature in the PGENv2 model with atmospheric CO_2_ concentration, $C_{a}=40$ Pa; absorbed short‐wave radiation $I_{\mathrm{abs}}=500$ W m^−2^; aerodynamic resistance to water vapour, $r_{a}=10$ s m^−1^; atmospheric vapour pressure, e=500 Pa. Within the white shaded region, stomatal conductance and photosynthesis are coupled ($dg_{\mathrm{sc}}/\mathrm{dA}>0$). Within the grey shaded region stomatal conductance and photosynthesis are decoupled ($dg_{\mathrm{sc}}/\mathrm{dA}<0$). Within the purple shaded region stomata are closed.

We also examined the role that soil moisture plays in the predicted decoupling. Figure 4 shows how the critical air temperature at which stomatal conductance decouples from photosynthesis depends on soil water potential. We define decoupling here as when the gradient of stomatal conductance with respect to photosynthesis becomes less than zero. The interface between the white and grey shaded regions of Figure 4 represents this critical temperature and depends on soil water potential. Specifically, as the soil becomes drier (i.e., the soil water potential decreases), this critical air temperature increases. This indicates a reduced ability of the plant to employ the thermoregulation strategy as soil moisture availability decreases. Similarly the interface of the grey and purple shaded regions in Figure 4 represents the critical air temperature at which the cost of water loss outweighs the benefit of evaporative cooling and stomata close. This too depends on soil water potential and we find that as water availability declines, this threshold temperature decreases. In effect this means that in the presence of increased water availability, our theory predicts that stomata are able to remain open at greater air temperatures. Conversely, when it is drier, the stomata shut earlier (i.e., at lower leaf temperatures) in order to conserve water. Overall the temperature window at which our model predicts decoupling is strongly dependent on soil water availability.

### 3.2 Modelling Stomatal Decoupling In Situ and Under Laboratory Conditions

**FIGURE 5:**
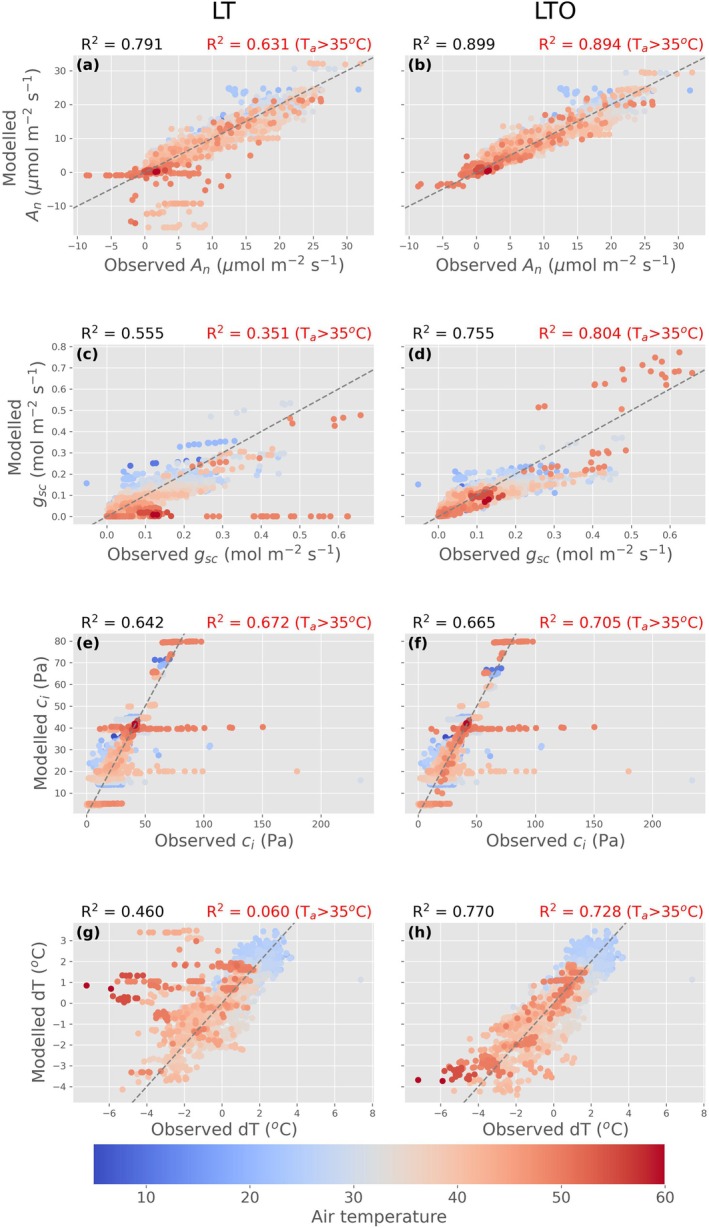
A comparison of observed and predicted photosynthesis (A, a, b); stomatal conductance to CO_2_ ($g_{\mathrm{sc}}$, c, d); internal leaf CO_2_ partial pressure ($c_{i}$, e, f); and the difference between leaf and air temperature (dT, g, h). Predictions are from the simplified PGENv2 model using the ‘Leaf Temperature’ (LT) approach to the leaf energy balance in the left column (panels a, c, e, g), and the ‘Leaf Temperature within Optimisation’ (LTO) in the right column (panels b, d, f, h). Observed gas exchange data were collated from the literature and represent 38 species from a range of environments. The *R* ^2^ values between model predictions and observations across the entire observed air temperature range for each variable is given above each panel in black. The *R* ^2^ values for each variable, for only the data corresponding to air temperatures greater than 35°C are given above each panel in red.

To evaluate the ability of the model to capture real examples of stomatal decoupling under high temperatures, we compared the predicted photosynthesis, stomatal conductance to CO_2_, and internal leaf CO_2_ partial pressure from the ‘LTO’ and ‘LT’ versions of the model to observed data (Figure 5) for 38 species across a range of environmental conditions. In general, we found that the ‘LTO’ model version captured the observed leaf gas exchange with higher accuracy that the ‘LT’ version. The *R* ^2^ of predicted by observed photosynthesis increased from 0.791 in the ‘LT’ model to 0.899 in the ‘LTO’ model. This improvement was more notable at higher temperatures (air temperatures greater than 35°C) where the *R* ^2^ increased from 0.631 to 0.894 for the ‘LT’ and ‘LTO’ models respectively. Similarly, the ‘LTO’ model made improvements to the predictions of stomatal conductance and internal leaf CO_2_ relative to the ‘LT’ model, where the *R* ^2^ values in the ‘LT’ model increased from 0.555 and 0.642 for stomatal conductance and internal leaf CO_2_ respectively to 0.755 and 0.665 in the ‘LTO’ model. Again the improvement was more notable at high temperatures where the *R* ^2^ values for $g_{\mathrm{sc}}$ and $c_{i}$ in the ‘LT’ model increased from 0.351 and 0.672 to 0.804 and 0.705 in the ‘LTO’ model.

The most notable improvements of the ‘LTO’ model compared to the ‘LT’ model however, were in the predictions of leaf temperature. In general both models captured the difference between leaf and air temperature well at low air temperatures. However, at high air temperatures, due to the lack of increased stomatal aperture, the ‘LT’ model failed to accurately capture observed dT values with an *R* ^2^ value of 0.060 ($T_{a}>35^{o}C$). The ‘LTO’ model on the other hand was significantly more capable at predicting the larger differences in leaf and air temperature that were seen at under extreme heat with a high temperature *R* ^2^ value of 0.728. This resulted in an *R* ^2^ value of 0.770 across the entire temperature range compared to the equivalent value of 0.460 from the ‘LT’ model. While the changes in predicted stomatal conductance at high temperatures were often relatively small (Figure S3; Table 2), these resulted in large differences in predicted leaf temperature due to the high sensitivity of transpiration to stomatal conductance when VPD is high.

**TABLE 2:** Species used for model validation.

Species	References	Notes
*Adelphia platyrachis*	Slot and Winter (2017)	
*Amphilophium paniculatum*	Slot and Winter (2017)	
*Aristolochia tonduzii*	Slot and Winter (2017)	
*Astronium graveolens*	Slot and Winter (2017)	
*Bonamia trichantha*	Slot and Winter (2017)	
*Brosimum utile*	Slot and Winter (2017)	
*Carapa guianensis*	Slot and Winter (2017)	
*Calligonum arborescens*	Feng et al. (2023)	Measurements taken under both well watered and drought conditions
*Calophyllum longifolium*	Slot et al. (2016)	Seedlings. Measurements made at both high (21%) and low (2%) oxygen
*Cecropia peltata*	Slot and Winter (2017)	
*Cordia bicolor*	Slot and Winter (2017)	
*Clethra fagifolia*	Taylor (2026)	
*Doliocarpus major*	Slot and Winter (2017)	
*Fagus sylvatica*	Diao et al. (2024)	Constant VPD
*Ficus insipida*	Slot et al. (2016)	Seedlings. Measurements made at both high (21%) and low (2%) oxygen
*Garcinia madruno*	Slot and Winter (2017)	
*Guatteria dumetorum*	Slot and Winter (2017)	
*Guatteria goudotiana*	Taylor (2026)	
*Haloxylon ammodendron*	Feng et al. (2023)	Measurements taken under both well watered and drought conditions
*Heisteria scandens*	Slot and Winter (2017)	
*Ilex laurina*	Taylor (2026)	
*Luehea seemannii*	Slot and Winter (2017)	
*Manilkara bidentata*	Slot and Winter (2017)	
*Miconia minutiflora*	Slot and Winter (2017)	
*Nectandra cuspidata*	Slot and Winter (2017)	
*Ochroma pyramidale*	Slot et al. (2016)	Seedlings. Measurements made at both high (21%) and low (2%) oxygen
*Passiflora vitifolia*	Slot and Winter (2017)	
*Pinus taeda*	Urban et al. (2017)	Experiments performed under dry, wet, and high CO_2_
*Populus deltoides*	Urban et al. (2017)	Experiments performed under dry, wet, and high CO_2_
*Picea abies*	Diao et al. (2024)	Constant VPD
*Quercus petraea*	Diao et al. (2024)	Constant VPD
*Schefflera morototoni*	Diao et al. (2024)	Constant VPD
*Serjania mexicana*	Slot and Winter (2017)	
*Spondias mombin*	Slot and Winter (2017)	
*Tilia cordata*	Diao et al. (2024)	Constant VPD
*Tocoyena pittieri*	Slot and Winter (2017)	
*Vantanea depleta*	Slot and Winter (2017)	
*Virola multiflora*	Slot and Winter (2017)	

Finally despite the uncertainty in the fitted photosynthetic parameters which was a result of low data availability and high parameter interdependency, we found that decoupling was a robust result across the posterior distribution of potential photosynthetic parameter values. Figure S5 shows the mean and standard deviation of predicted gas exchange for three species, using a random sample of photosynthetic parameters from across the posterior distribution. While changing the photosynthetic parameters did result in changes in predicted stomatal conductance, in particular at high temperatures, in general the model still predicted decoupling for all combinations of photosynthetic parameters that could reasonably describe the observed photosynthesis data.

### 3.3 The Impacts of Evaporative Cooling on the Medlyn Stomatal Conductance Model

When the effect of stomatal conductance on leaf temperature was accounted for in the derivation of the $g_{1}$ parameter, the form of Equation (41) remained unchanged, however, the updated $g_{1}$ parameter (which we denote here as $g_{1}^{*}$) was found to be approximated by the following expression:

(43) $$g_{1}^{*}\propto \sqrt{\frac{\Gamma^{*}\lambda }{\left(1-\xi \lambda \right)}}$$

Here ξ represents the coupling strength of the evaporative cooling feedback and is given mathematically by:

(44) $$\xi =\frac{1}{D}\frac{\partial A}{\partial T_{l}}\frac{\partial T_{l}}{\partial g_{\mathrm{sv}}}$$

where D is vapour pressure deficit; $\partial A/\partial T_{l}$ is the partial derivative of leaf photosynthesis with respect to leaf temperature ($T_{l}$); and $\partial T_{l}/\partial g_{\mathrm{sv}}$ is the partial derivative of leaf temperature with respect to stomatal conductance to water vapour.

We first note that if we revert to the traditional optimisation approach where the leaf energy balance is not accounted for, then leaf temperature does not depend on stomatal conductance and the last term ($\partial T_{l}/\partial g_{\mathrm{sv}}$) in this equation is zero. In this case the ξ becomes zero and Equation (43) reverts back to the original expression derived in Medlyn et al. (2011) (Equation 42). It should also be noted that this expression is an approximation (Notes S1) and does not exactly describe how $g_{1}$ changes with temperature. However, it is useful here to gain a mathematical understand how implementing the leaf energy balance impacts $g_{1}$ at least to first order, which we describe below.

**FIGURE 6:**
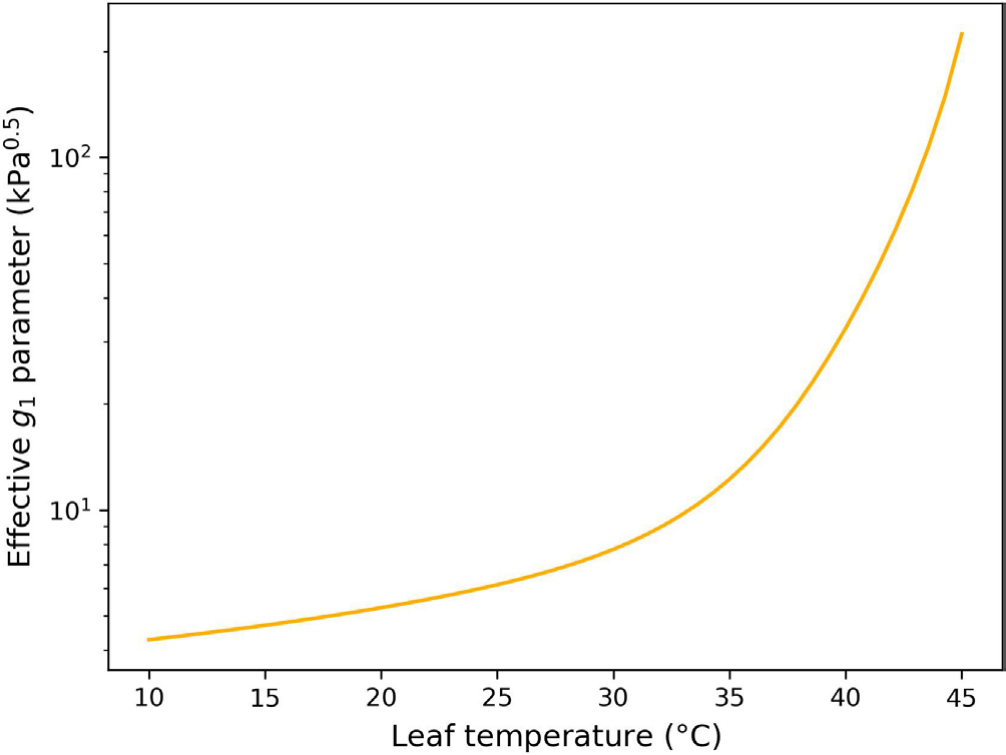
The effective $g_{1}$ parameter for the simplified PGENvn2 model against predicted leaf temperature for the ‘Leaf Temperature within Optimisation’ (LTO) approach to the leaf energy balance. The simulation was performed with atmospheric CO_2_ concentration, $C_{a}=40$ Pa; soil water potential, $\psi_{s}=-0.1$ MPa; absorbed short‐wave radiation $I_{\mathrm{abs}}=500$ W m^−2^; aerodynamic resistance to water vapour, $r_{a}=10$ s m^−1^; and atmospheric vapour pressure, e=500 Pa.

This new approximation tells us that the $g_{1}$ parameter cannot be considered constant. In particular, due to the dependence of the slope of photosynthesis with respect to leaf temperature (the partial derivative of A with respect to $T_{l}$ in Equation 44) on temperature we expect $g_{1}$ to change with temperature. Specifically when leaf temperature is less than the thermal optimum this slope is positive. Given that D is always positive, and leaf temperature decreases with increasing stomatal conductance (i.e., the partial derivative of $T_{l}$ with respect to $g_{\mathrm{sc}}$ is negative), this means that when leaf temperature is below the thermal optimum for A, the feedback strength (ξ) is negative. This results in a smaller value of $g_{1}$ than otherwise expected. Conversely, when leaf temperature exceeds the thermal optimum of A, the partial derivative of A with respect to $T_{l}$ becomes negative which results in an increased value of $g_{1}$. For a smooth dependence of photosynthesis with respect to leaf temperature, we therefore expect $g_{1}$ to strongly increase with leaf temperature. Indeed this is what we found when we calculated the effective $g_{1}$ parameter for the PGENv2 model (Figure 6).

## 4 Discussion

As the climate changes, large parts of the globe are expected to experience increases in the frequency and severity of heat extremes (Domeisen et al. 2023). The decoupling of photosynthesis and stomatal conductance will have a large impact on the fluxes of water and energy between the land and atmosphere. At present, predictions of stomatal conductance and photosynthesis are tightly linked in land surface models. Simulations of the climate will therefore fail to capture the partitioning of sensible and latent heat fluxes and the rate of transpiration in hot regions now and in the future. By coupling the leaf energy balance to a simple stomatal optimisation model, it is possible to capture the decoupling of stomatal conductance and photosynthesis at high temperatures (Friend 1995). This suggests that the decoupling of stomatal conductance and photosynthesis is consistent with the hypothesis that when temperatures exceed the thermal optimum for photosynthesis, leaves actively prioritise evaporative cooling over water retention (Friend 1995). However, we show here that calculating the leaf energy balance outside the optimisation scheme does not produce the same results, even when iterating to convergence as is commonly done in Earth System Models. Given the significant divergence in predicted stomatal conductance under high temperatures between these two approaches, correctly accounting for the leaf energy balance is a necessity for the development of future stomatal optimisation models.

The dependence of predicted stomatal decoupling on soil water potential in our model suggests a fine balance between the benefit of evaporative cooling and the cost of excessive water loss. However, it is not clear how decoupling depends on water availability in the real world. Intuition would suggest that a sufficient water supply is required to maintain transpiration if the plant is to benefit from evaporative cooling (Diao et al. 2024). Indeed, Feng et al. (2023), Slot et al. (2016), and Urban et al. (2017) all found evidence that decoupling was less pronounced during drought. Similarly, a number of studies that find significant evidence of decoupling (Drake et al. 2018; Krich et al. 2022; Diao et al. 2024; Chen et al. 2025), all assume that the measured plants had non‐limiting access to water. However, work by Marchin et al. (2023) found evidence that decoupling was more pronounced in plants with severely limited access to water than in those that were well watered. Similarly, Marchin et al. (2022) found a relative reduction in stomatal conductance in well‐watered plants during an artificial heat wave, but a relative increase in plants experiencing water stress. These contrasting findings point to a complex interaction between stomatal decoupling and available water that our model cannot currently capture. The PGENv2 model accounts for the impact of water availability on stomatal conductance through a linear cost function which represents the dependence of plant growth on leaf water potential (Fricke 2017). However, it is well established that many plants maintain stomatal conductance, even after growth has been limited (Fatichi et al. 2014). These species likely prioritise carbon acquisition over maintaining cell turgor pressure and will close stomata only to avoid hydraulic failure. This strategy is captured by optimisation models that use a sigmoidal cost function associated with xylem cavitation (e.g., Sperry et al. 2017; Eller et al. 2018, 2020). To begin to understand the impact of the hydraulic cost function in our model, we repeated the analysis in Figure 4, replacing the linear cost function of PGEN with a non‐linear vulnerability curve associated with xylem conductance as described by Eller et al. (2020). The impact of this change on predicted decoupling was profound and we saw a reversal of the predicted drought response, with stomatal decoupling occurring more readily in dryer soils (Figure S6). This was due to reduced relative cost of opening stomata (characterised by the shallower gradient of the vulnerability curve) when significant amounts of xylem conductance had already been lost. In other words when the plant was extremely drought stressed, there was little risk of further losses in xylem (since most of it was already embolised) and hence decoupling occurred more readily. This may explain the variation in drought responses of decoupling events seen across the literature, and suggests that more conservative plants (i.e., more isohydric) that close their stomata quickly during drought (i.e., like those that are represented by PGENv2) should experience less decoupling under drought. Meanwhile plants that take greater hydraulic risks during drought (as seen when optimising xylem conductance instead of cell turgor) may experience greater decoupling during drought. Testing this hypothesis, and more generally understanding how prolonged stomatal opening depends on water availability, and how this varies across different water‐use strategies should be a priority. Over time, sustained stomatal decoupling may bring plants closer to dangerous levels of water scarcity putting them at risk of hydraulic failure and mortality which could have significant impacts on the global carbon and water cycles. Plant responses to drought are already poorly captured by models (Powell et al. 2013) although to what extent the missing thermoregulation process contributes to this model uncertainty is unclear.

The Medlyn stomatal conductance model is popular amongst land surface modellers. It is parsimonious, has an analytical form, and the parameters can be easily estimated from available data. However, it is not consistent with stomatal decoupling as the constant $g_{1}$ parameter explicitly couples conductance with photosynthesis. Increasingly, the assumption that $g_{1}$ is constant is being questioned (Wu et al. 2020; Potkay et al. 2025; Lin et al. 2015; Franks et al. 2024; Cheesman et al. 2026) and there are some well established reasons why we should expect $g_{1}$ to vary. These include the temperature response of the CO_2_ compensation point ($\Gamma^{*}$) and variation in the marginal water cost of carbon (λ) (Medlyn et al. 2011), water availability (Franks et al. 2024), as well as less studied effects including the temperature dependence of the viscosity of water (but see Diao et al. (2024); Cheesman et al. (2026)). Here we have shown that when the leaf energy balance is included in the Medlyn model, an additional source of variation in the $g_{1}$ parameter is introduced (Figure 6). This variation is comparatively large and occurs over rapid timescales and so clearly cannot be ignored when considering high temperature responses. Our new formulation for $g_{1}$ (Equation 43) provides insight into the origin of this variation, however, we should stress that it is an approximation, and can diverge from the true value of $g_{1}$, in particular when the evaporative cooling feedback strength (ξ) is large. As a result, our reformulation of the $g_{1}$ parameter should not be seen as a convenient upgrade, but instead simply as a demonstration that significant variation in $g_{1}$ should be expected at high temperatures. It is often difficult to find exact analytical solutions to stomatal optimisation models without some simplifying assumptions (e.g., Eller et al. 2020), and the addition of the leaf energy balance further increases this complexity. Solving these models numerically can be computationally expensive (Jones et al. 2022), and is in part why analytical models like that of Medlyn et al. (2011) are attractive to earth system modellers. However, there are a growing number of processes that are considered important for predicting stomatal conductance. As well as leaf temperature, the role of carbohydrate storage and nutrient availability which can directly affect plant growth and hence the balance of costs and benefits of opening stomata are thought to be important (Potkay et al. 2021; Potkay and Feng 2023; Blonder et al. 2023). Finding simple analytical forms that can account for all these feedbacks is unlikely. We therefore need a greater focus on the numerical methods with which optimisation models are solved so that these processes may be accounted for efficiently within global models (Jones et al. 2022).

Temperature is one of the most important drivers of plant behaviour. It influences almost all biological processes and as a result, alongside precipitation, is one of the key determinants of plant functional distributions across the globe (Reich et al. 2014; Violle et al. 2014). Feedbacks within the earth system mean that small changes in the sensitivities of plant responses to temperature can have large effects on the global climate (Mercado et al. 2018; Huntingford et al. 2017; Cox et al. 2000). Accurately representing the correct responses to temperature is therefore crucial. Decoupling aside, accounting for the leaf energy balance in large scale models may significantly impact the way plants are predicted to respond to climate change. Representing distinct temperatures for the canopy and boundary layer is not common practice in Earth System Models, and yet it is well established that leaf temperature can differ by several degrees from air temperature (Still et al. 2022). The impact of this on carbon, water and energy fluxes can be large, especially over short timescales when the variation of processes like autotrophic respiration is dominated by its sensitivity to temperature (Heskel et al. 2016; Atkin et al. 2015; Jones et al. 2024). The long term temperature responses of both respiration and photosynthesis are ongoing areas of research but increasingly, their short term sensitivities to temperature are found not to hold over longer timescales (Wang et al. 2026; Cox et al. 2023; Kumarathunge et al. 2019). As new understanding emerges, accurate predictions of leaf, and ultimately canopy temperatures will be needed in ESMs so that new processes can be implemented and captured correctly. Further work is required to incorporate the leaf energy balance in ESMs and allow temperature differences within the land surface to be represented. Such an effort may improve the predictions of numerous processes within terrestrial ecosystems, as well as allowing the important high temperature response of stomata to be captured during heat extremes.

## 5 Conclusions

Recent observational data shows evidence that stomatal conductance and photosynthesis can decouple at high temperatures, with stomatal conductance continuing to increase even though photosynthesis declines at high temperatures (De Kauwe et al. 2019; Diao et al. 2024). This behaviour is seemingly at odds with the semi‐empirical (Ball et al. 1987; Leuning 1995) and stomatal optimisation models Cowan and Farquhar (1977), Medlyn et al. (2011), Eller et al. (2020) that are embedded in our global climate and Earth System models. Stomatal decoupling remains consistent with optimal stomatal behaviour if the impact of stomatal conductance on leaf surface temperature is included within optimisation models (Friend 1995; Sabot et al. 2020; Sicangco et al. 2026). However, here we have demonstrated that this is only true if leaf temperature is calculated in parallel with optimal stomatal conductance, and not as part of a fixed point iteration, as is commonly done in Earth System models. This difference is subtle but has important implications for the high temperature response of stomata. We found that by accounting for leaf temperature in this way, we could more accurately capture measured leaf gas exchange and leaf‐to‐air temperature differences across a range of plant species. We conclude that it is critically important for global models to include a fully integrated leaf energy balance if they are to reproduce the observed photosynthesis–stomatal decoupling at high temperatures.

## Author Contributions

**Lucas A. Cernusak:** writing – review and editing, methodology. **Alexander W. Cheesman:** writing – review and editing, methodology. **Andrew D. Friend:** methodology, writing – review and editing. **Xiaolong Feng:** writing – review and editing, data curation. **Simon R. G. Jones:** investigation, writing – original draft, methodology, validation, visualization, writing – review and editing. **Peter J. Franks:** writing – review and editing, methodology. **Tyeen Taylor:** writing – review and editing, data curation. **Haoyu Diao:** methodology, writing – review and editing, data curation. **Martijn Slot:** writing – review and editing, data curation. **Lina M. Mercado:** methodology, writing – review and editing. **Georg Wohlfahrt:** conceptualization, writing – review and editing, methodology. **Josef Urban:** writing – review and editing, data curation. **Peter M. Cox:** conceptualization, writing – review and editing, methodology.

## Funding

This work was supported by Natural Environment Research Council, NE/X019055/1, NE/S015833/1, NE/X001172/1, NE/W004895/1, NE/W000199/1; Biotechnology and Biological Sciences Research Council, BB/V011588/1; HORIZON EUROPE Climate, Energy and Mobility, 101081193; Austrian Science Fund: PAT2661823, P35737; Human Frontier Science Program, RGP0016/2020; Australian Research Council, DP240101938; Bilateral project of the Czech Science Foundation, 25‐14626L and the Polish National Science Centre 2023/51/I/NZ9/01813.

## Conflicts of Interest

The authors declare no conflicts of interest.

## Supporting information


**Figure S1:** The corresponding change in atmospheric vapour pressure deficit (VPD) with atmospheric temperature, corresponding to the constant vapour pressure (e) used in Figures 2–4 of the main text.
**Figure S2:** The predicted response of (a) photosynthesis, (b) stomatal conductance to CO2, (c) the difference between leaf and atmospheric temperature, and (d) internal leaf CO2 partial pressure to changes in atmospheric temperature from the simplified PGEN model.
**Figure S3:** The response of photosynthesis (A, μmol m−2 s−1‐columns 1 + 5); stomatal conductance to CO2 (gsc, mol m−2 s−1‐columns 2 + 6); internal leaf CO2 partial pressure (ci, Pa—columns 3 + 7); and the difference between leaf and air temperature (dT, °C—columns 4 + 8) to air temperature for the 38 species described in Table 2 of the main text.
**Figure S4:** A comparison of fitted vs. observed photosynthesis (A, μmol m−2 s−1). The fitted photosynthesis is calculated using the parameters given in Table S1 and the equations for leaf photosynthesis in terms of total conductance to CO2 given in Notes S1.
**Figure S5:** The impact of parameter uncertainty within the Farquhar photosynthesis model on predicted photosynthesis (A, μmol m−2 s−1); stomatal conductance to CO2 (gsc); internal leaf CO2 partial pressure (ci, Pa); and the difference between leaf and air temperature (dT).
**Figure S6:** The dependence of decoupling on soil water potential and air temperature when using a sigmoidal hydraulic cost function.
**Table S1:** Fitted parameters and their standard error from the non‐linear least squares fitting of photosynthesis, the leaf energy balance, and the critical stomatal conductance. Where measure values have been used in place of fitted parameters is noted.
**Notes S1** Deriving the modified Medlyn g1 parameter.

## Data Availability

The processed data used to produce the figures are available at https://doi.org/10.5281/zenodo.20669500. The raw data is available at https://doi.org/10.5281/zenodo.20669292.
