# Supplementary material for: Stomatal Decoupling From Photosynthesis Under High Temperatures Is Consistent With Stomatal Optimisation
Source: Glob Chang Biol. 2026 Jul 2;32(7):e70972. doi: 10.1111/gcb.70972 (PMC13324414; doi:10.1111/gcb.70972)
Supplement: Supplementary file 1 — Figure S1: The corresponding change in atmospheric vapour pressure deficit (VPD) with atmospheric temperature, corresponding to the constant vapour pressure (e) used in Figures 2–4 of the main text. Figure S2: The predicted response of (a) photosynthesis, (b) stomatal conductance to CO2, (c) the difference between leaf and atmospheric temperature, and (d) internal leaf CO2 partial pressure to changes in atmospheric temperature from the simplified PGEN model. Figure S3: The response of photosynthesis (A, μmol m−2 s−1‐columns 1 + 5); stomatal conductance to CO2 (gsc, mol m−2 s−1‐columns 2 + 6); internal leaf CO2 partial pressure (ci, Pa—columns 3 + 7); and the difference between leaf and air temperature (dT, °C—columns 4 + 8) to air temperature for the 38 species described in Table 2 of the main text. Figure S4: A comparison of fitted vs. observed photosynthesis (A, μmol m−2 s−1). The fitted photosynthesis is calculated using the parameters given in Table S1 and the equations for leaf photosynthesis in terms of total conductance to CO2 given in Notes S1. Figure S5: The impact of parameter uncertainty within the Farquhar photosynthesis model on predicted photosynthesis (A, μmol m−2 s−1); stomatal conductance to CO2 (gsc); internal leaf CO2 partial pressure (ci, Pa); and the difference between leaf and air temperature (dT). Figure S6: The dependence of decoupling on soil water potential and air temperature when using a sigmoidal hydraulic cost function. Table S1: Fitted parameters and their standard error from the non‐linear least squares fitting of photosynthesis, the leaf energy balance, and the critical stomatal conductance. Where measure values have been used in place of fitted parameters is noted. Notes S1 Deriving the modified Medlyn g1 parameter. [file GCB-32-e70972-s001.docx]

**Supplementary Material**

**Contents**

**Supplementary Figure S1** The dependence of VPD on air temperature

**Supplementary Figure S2** Predicted temperature responses under constant VPD

**Supplementary Figure S3** Observed and predicted gas exchange per species

**Supplementary Figure S4** Observed vs fitted photosynthesis.

**Supplementary Figure S5** The impact of photosynthetic parameter uncertainty

**Supplementary Figure S6** The drought response using a sigmoidal cost function

**Supplementary Table S1** The results of non-linear least squares.

**Supplementary Notes S1** Deriving the modified Medlyn g1 parameter

**Supplementary Figure S1**

**
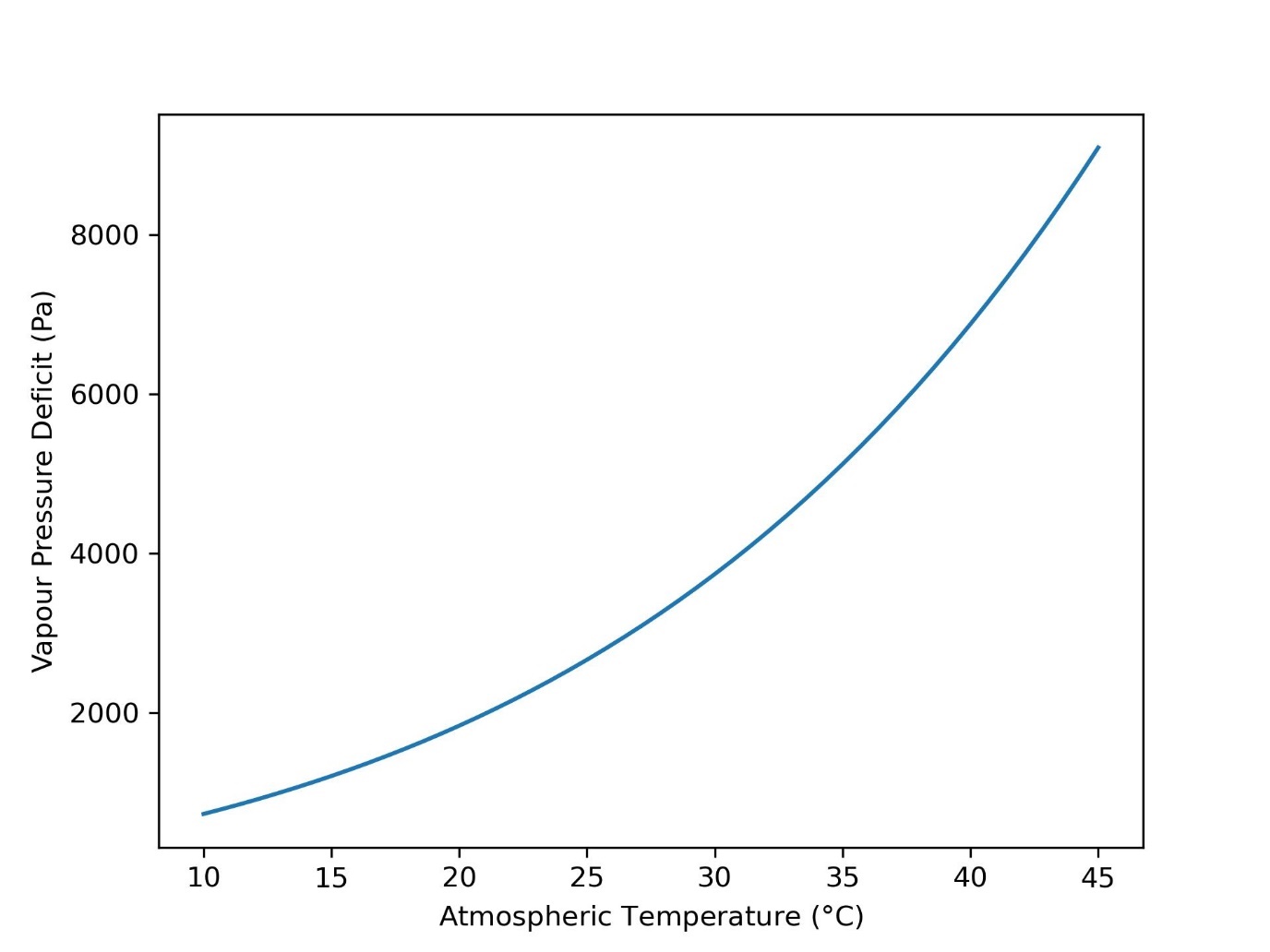
**

Figure S1 The corresponding change in atmospheric vapour pressure deficit (VPD) with atmospheric temperature, corresponding to the constant vapour pressure ($e$) used in Figures 2, 3 and 4 of the main text.

**Supplementary Figure S2**


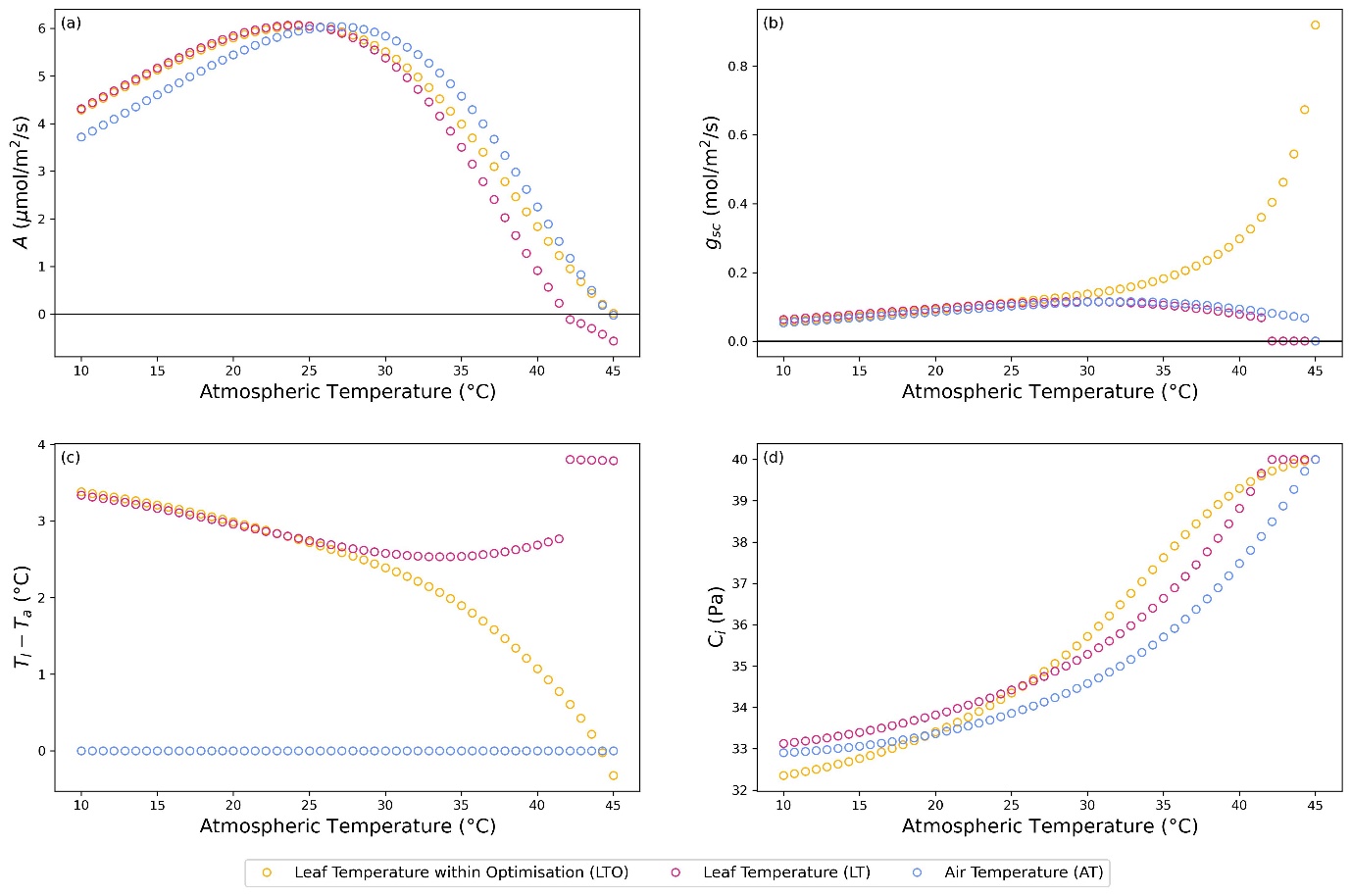


Figure S2 The predicted response of (a) photosynthesis, (b) stomatal conductance to CO_2_, (c) the difference between leaf and atmospheric temperature, and (d) internal leaf CO_2_ partial pressure to changes in atmospheric temperature from the simplified PGEN model. Yellow markers show the response using the ‘Leaf Temperature within Optimisation' (LTO) approach to the leaf energy balance, pink markers show the response using the ‘Leaf Temperature' (LT) approach, and blue markers show the response with the ‘Air Temperature' (AT) approach. The simulations are conducted with: atmospheric CO_2_ concentration, $C_{a}$=40Pa; soil water potential, $\psi_{s}$= -0.1MPa; incoming short-wave radiation $I_{s}$ = 500 W m^-2^; aerodynamic resistance to water vapour, $r_{a}$ = 10 s m^-1^; vapour pressure deficit, D = 1000Pa.

**Supplementary Figure S3**

**
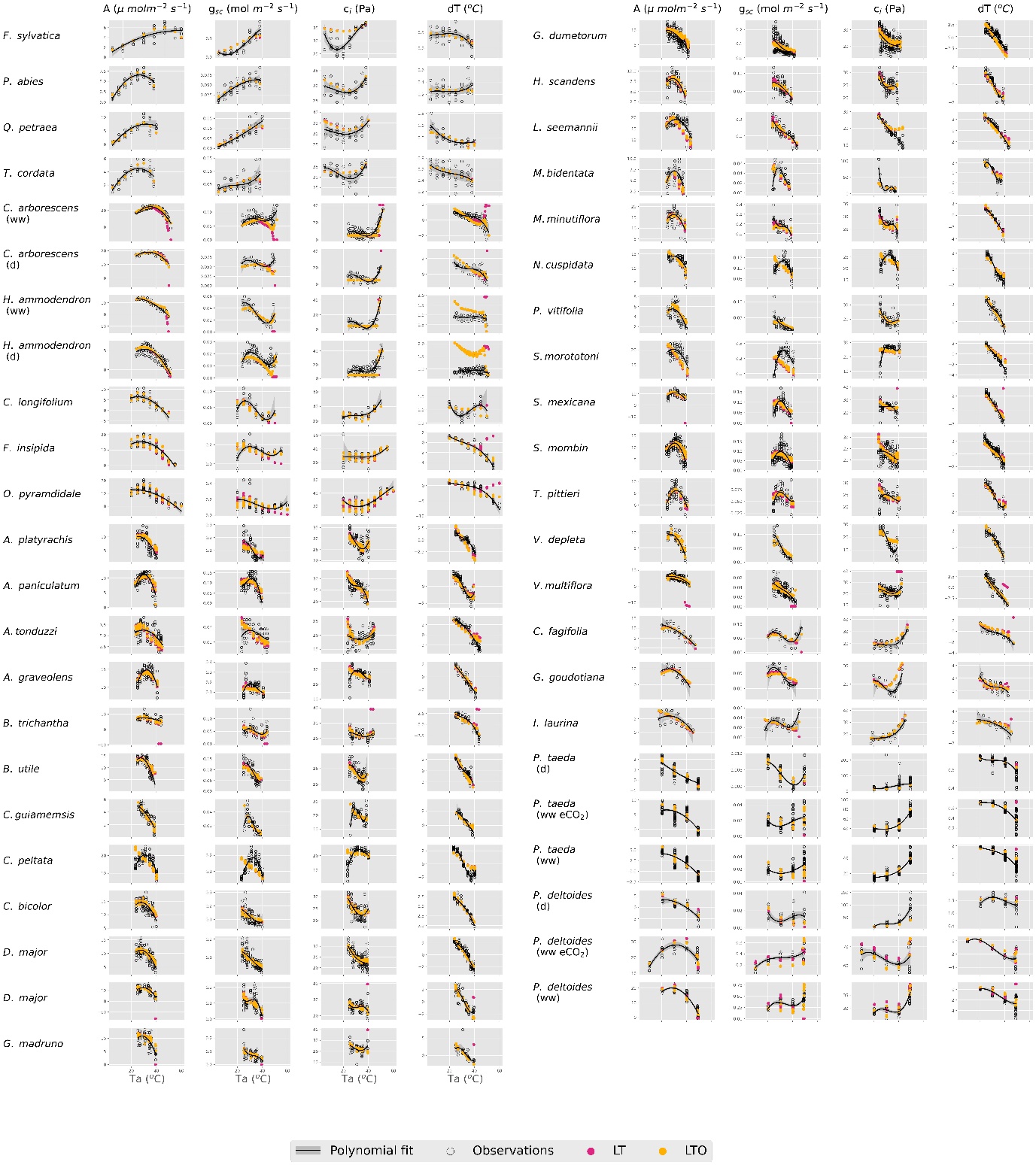
**

Figure S3 The response of photosynthesis (A, $\mu$mol m^-2^ s^-1^- columns 1 + 5); stomatal conductance to CO_2_ ($g_{sc}$, mol m^-2^ s^-1^- columns 2 + 6); internal leaf CO2 partial pressure ($c_{i}$, Pa – columns 3 + 7); and the difference between leaf and air temperature ($dT$, ^o^C – columns 4 + 8) to air temperature for the 38 species described in Table 2 of the main text. Species that have been grown in well-watered (ww), droughted (d) and elevated CO_2_ (eCO_2_) are noted below the species names. Polynomials are fitted to the observations (empty black circles) and plotted (black line) to visualise the general observed trends. Predictions by the simplified PGEN model using the ‘Leaf Temperature within Optimisation' (LTO) approach to the leaf energy balance are shown in yellow, and predictions using the ‘Leaf Temperature' (LT) approach are shown in pink.

**Supplementary Figure S4**

**
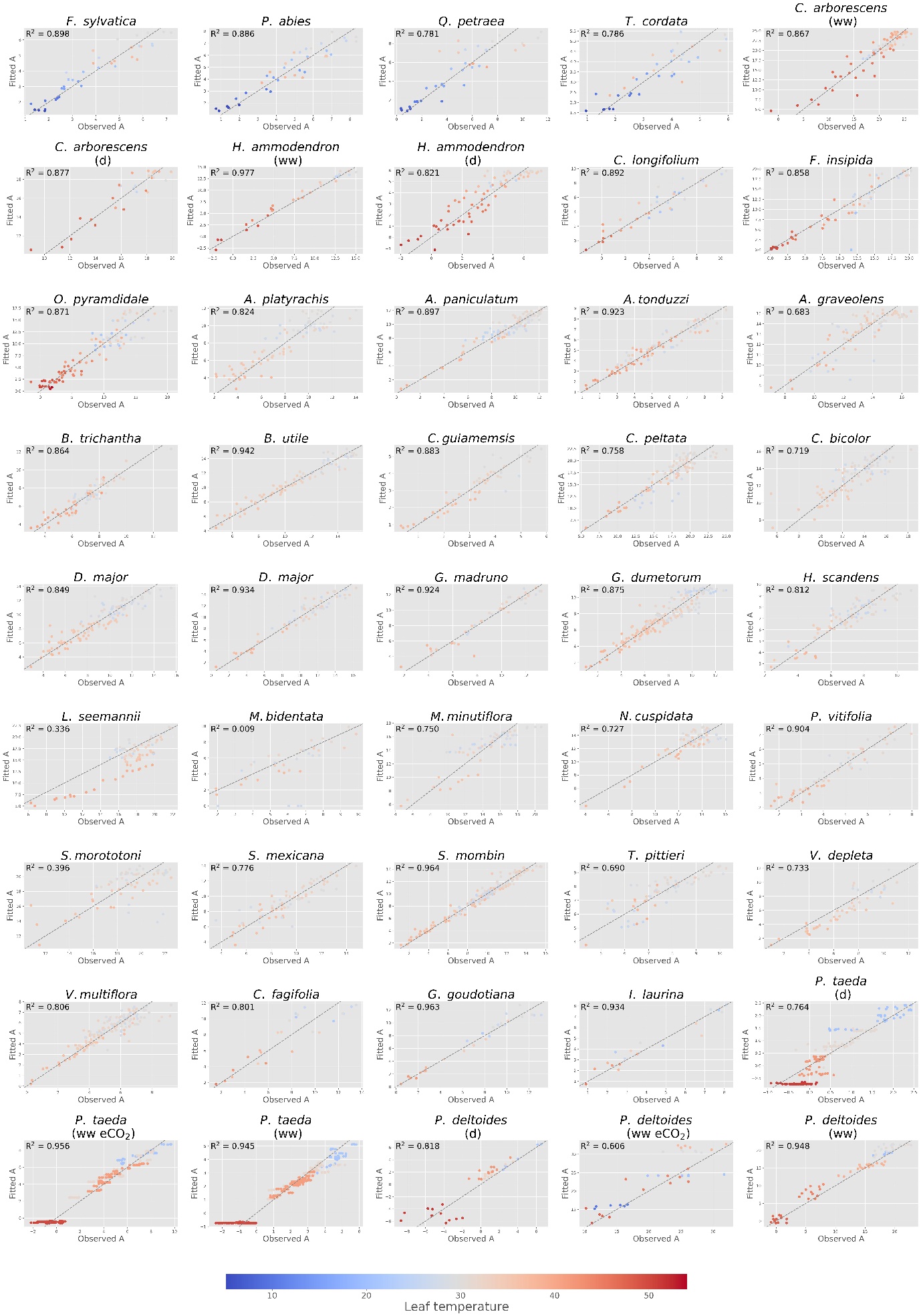
**

Figure S4 A comparison of fitted vs observed photosynthesis (A, $\mu$mol m^-2^ s^-1^). The fitted photosynthesis is calculated using the parameters given in Table S1 and the equations for leaf photosynthesis in terms of total conductance to CO_2_ given in Supplementary notes S1.

**Supplementary Figure S5**

**
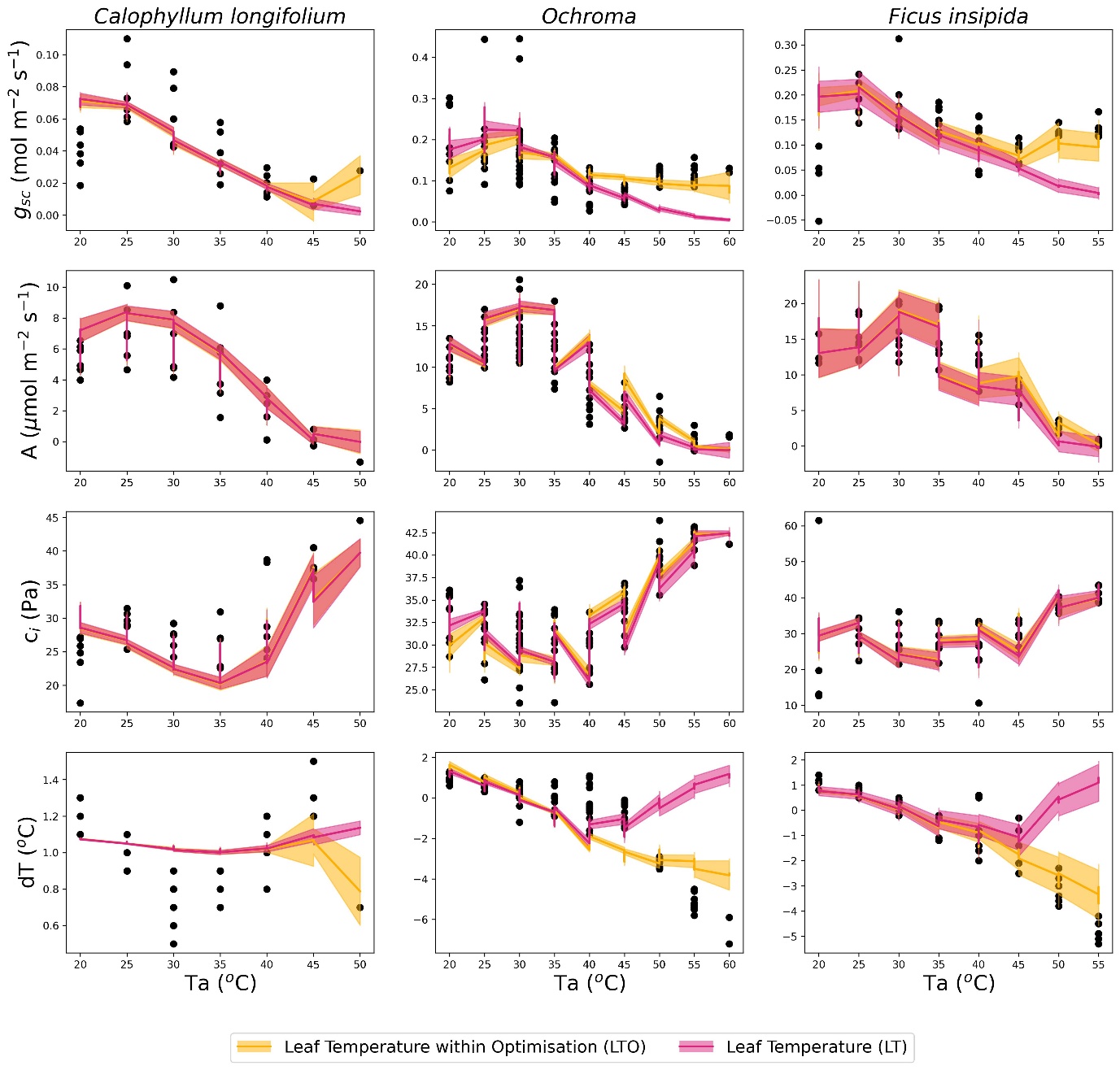
**

Figure S5 The impact of parameter uncertainty within the Farquhar photosynthesis model on predicted photosynthesis (A, $\mu$mol m^-2^ s^-1^); stomatal conductance to CO_2_ ($g_{sc}$); internal leaf CO2 partial pressure ($c_{i}$, Pa); and the difference between leaf and air temperature ($dT$). The solid lines represent the mean prediction using a random sample of 100 sets of parameters from the posterior distribution of photosynthetic parameters determined using a Markov-Chain Monte Carlo (MCMC) sampler. The shaded regions represent 1 standard deviation of the random sample from the mean.

**Supplementary Figure S6**

**
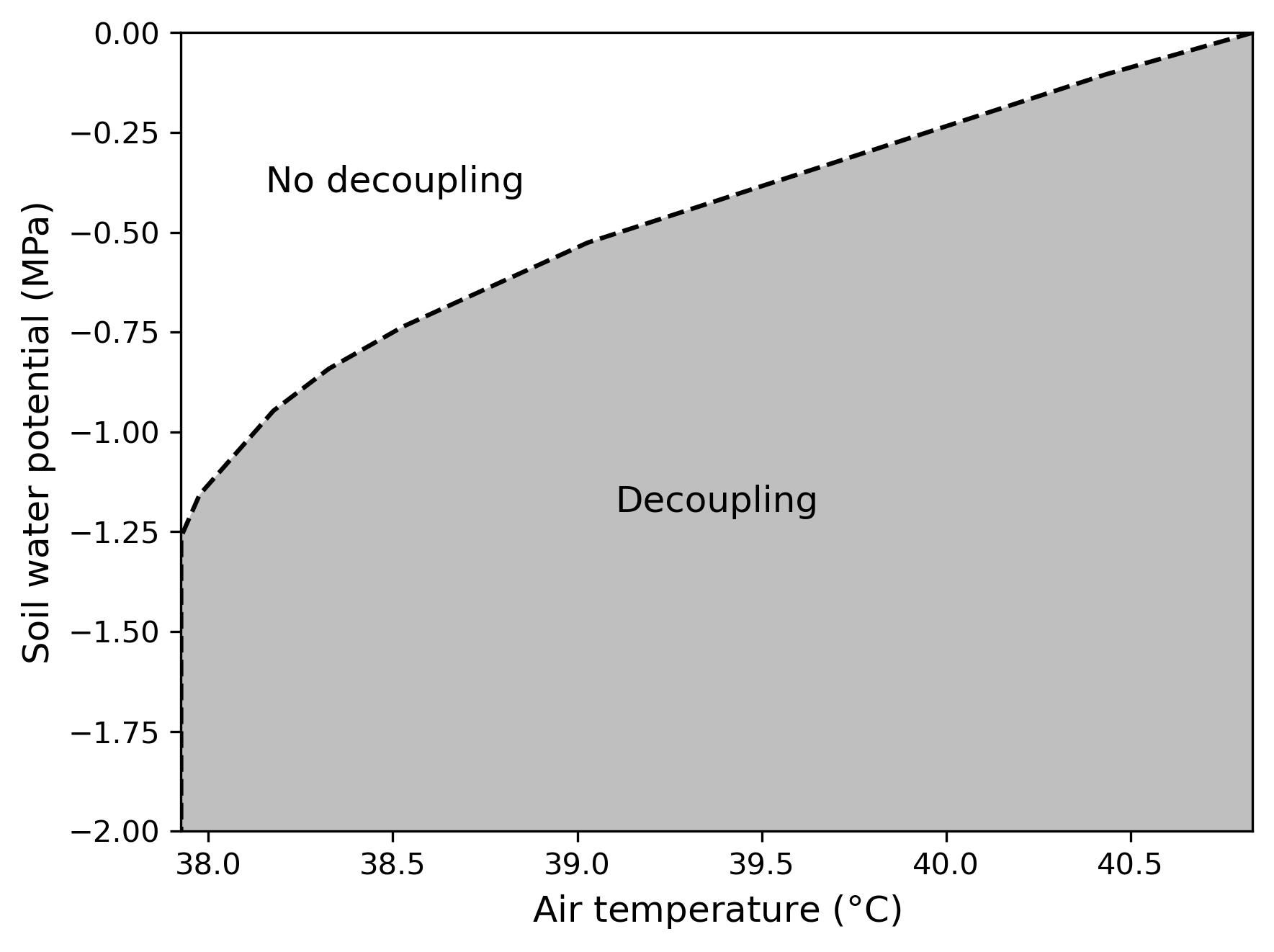
**

Figure S6 The dependence of decoupling on soil water potential and air temperature when using a sigmoidal hydraulic cost function. Environmental conditions are atmospheric CO_2_ concentration, $C_{a} = 40Pa$; absorbed short-wave radiation, $I_{abs}=500Wm^{-2}$; aerodynamic resistance to water vapour, $r_{a}=10 sm^{-1};$ atmospheric vapour pressure$, e=500Pa$. Within the white shaded region, stomatal conductance and photosynthesis are coupled ($dg_{sc}/dA>0$). Within the grey shaded region stomatal conductance and photosynthesis are decoupled ($dg_{sc}/dA<0$).

**Supplementary Table S1**

| Species | $\boldsymbol{E}_{\boldsymbol{a}}$ $\boldsymbol{(\times}{10}^{3}\boldsymbol{)}$ | $\boldsymbol{\eta}$ | $\boldsymbol{T}_{\boldsymbol{opt}}$ | $\boldsymbol{vcma}\boldsymbol{x}_{\boldsymbol{25}}$ | $\boldsymbol{jma}\boldsymbol{x}_{\boldsymbol{25}}$ | $\boldsymbol{f}_{\boldsymbol{d}}$ | $\boldsymbol{q}_{\boldsymbol{10}}$ | $\boldsymbol{\beta}_{\boldsymbol{LTO}}$ | $\boldsymbol{\beta}_{\boldsymbol{LT}}$ | $\boldsymbol{r}_{\boldsymbol{a}}$ | $\boldsymbol{I}_{\boldsymbol{s}}$ |
| --- | --- | --- | --- | --- | --- | --- | --- | --- | --- | --- | --- |
| *F. sylvatica* | 62.1 ± 13.5 | 2.14 ± 1.49 | 35 ± 7.64 | 6.1e-05 ± 6.24e-05 | 5.39e-05 ± 2.87e-05 | measured | measured | 0.00687 ± 0.000786 | 0.00677 ± 0.000899 | 2.48 ± 0.452 | 297 ± 27.3 |
| *P. abies* | 86.5 ± 17 | 2.36 ± 1.14 | 35 ± 1.02 | 5.81e-05 ± 4.87e-06 | 0.001 ± 0.0311 | measured | measured | 0.00178 ± 0.000122 | 0.00178 ± 0.000124 | 0.708 ± 0.693 | 500 ± 355 |
| *Q. petraea* | 98.6 ± 37.7 | 1.35 ± 0.982 | 35 ± 14 | 0.00011 ± 0.000301 | 7.55e-05 ± 6.45e-05 | measured | measured | 0.00514 ± 0.000512 | 0.0051 ± 0.000566 | 5.25 ± 0.942 | 240 ± 18.4 |
| *T. cordata* | 56.6 ± 14.7 | 4.72 ± 2.3 | 34.4 ± 1.81 | 3.91e-05 ± 7.71e-06 | 6.69e-05 ± 4.77e-05 | measured | measured | 0.00326 ± 0.000491 | 0.00332 ± 0.000551 | 2.95 ± 0.535 | 320 ± 32.1 |
| *C. arborescens (ww)* | 38.1 ± 41.2 | 3.15 ± 7.36 | 45.6 ± 39.5 | 3.75e-05 ± 0.00013 | N/A (C4) | 0.0562 ± 0.641 | 3 ± 12.4 | 0.00818 ± 7.72e-05 | 0.00681 ± 0.000133 | measured | measured |
| *C. arborescens (d)* | 16.8 ± 5.98 | 18.5 ± 51.3 | 46.1 ± 15.8 | 2.63e-05 ± 2.58e-06 | N/A (C4) | 0.0429 ± 0.206 | 2.98 ± 6.81 | 0.00619 ± 0.000106 | 0.00771 ± 0.00124 | measured | measured |
| *H. ammodendron (ww)* | 23.9 ± 24.4 | 31 ± 100 | 48.1 ± 6.05 | 3.11e-05 ± 4.72e-05 | N/A (C4) | 0.1 ± 0.307 | 2.51 ± 3.11 | 0.00265 ± 3.98e-05 | 0.00267 ± 0.000301 | measured | measured |
| *H. ammodendron (d)* | 106 ± 399 | 1.18 ± 3 | 34.7 ± 39.4 | 7.35e-06 ± 1.89e-05 | N/A (C4) | 0.1 ± 1.26 | 2.27 ± 8.32 | 0.00164 ± 1.24e-05 | 0.00154 ± 2.21e-05 | measured | measured |
| *C. longifolium* | 77.7 ± 19.6 | 3.36 ± 2.16 | 36.3 ± 2.22 | 4.29e-05 ± 1.05e-05 | 0.001 ± 0.0666 | 0.00266 ± 0.0151 | 2.97 ± 6.71 | 0.00272 ± 0.000131 | 0.00287 ± 0.00037 | 1.94 ± 0.651 | 681 ± 162 |
| *F. insipida* | 18.8 ± 15.6 | 27.1 ± 23.7 | 39.3 ± 2.75 | 0.000162 ± 7.65e-05 | 0.000118 ± 4.24e-05 | 1.29e-36 ± 0.00263 | 1.57 ± 0 | 0.0137 ± 0.00116 | 0.0127 ± 0.00195 | 13.4 ± 1.25 | 255 ± 10.4 |
| *O. pyramdidale* | 43.8 ± 10.5 | 4.55 ± 1.67 | 33.2 ± 2.03 | 0.000138 ± 0.000106 | 8.52e-05 ± 1.33e-05 | 1.67e-16 ± 0.0038 | 1.29 ± 0 | 0.017 ± 0.00127 | 0.0216 ± 0.00187 | 23.5 ± 2.34 | 246 ± 5.54 |
| *A. platyrachis* | 168 ± 90.4 | 2.09 ± 1.27 | 32.6 ± 1.04 | 7.43e-05 ± 3.83e-05 | 0.001 ± 0.114 | 1.07e-11 ± 0.0441 | 1 ± 0 | 0.00928 ± 0.000787 | 0.01 ± 0.000649 | 361 ± 57.3 | 175 ± 5.07 |
| *A. paniculatum* | 71.1 ± 91.2 | 17.9 ± 2.68e+05 | 43.6 ± 8.77e+04 | 0.000414 ± 2.74 | 0.000452 ± 2.99 | 0.0114 ± 75.6 | 1 ± 2.03 | 0.00723 ± 0.000112 | 0.00657 ± 0.000302 | 18.6 ± 2.05 | 223 ± 4.44 |
| *A. tonduzzi* | 143 ± 354 | 2.54 ± 20.5 | 40.2 ± 40.3 | 0.001 ± 0.00685 | 0.000489 ± 0.00352 | 0.00137 ± 0.017 | 2.58 ± 27.3 | 0.00233 ± 0.000138 | 0.00215 ± 0.000137 | 74.9 ± 9.58 | 194 ± 3.35 |
| *A. graveolens* | 124 ± 281 | 1.17 ± 1.88 | 40.3 ± 24.7 | 0.001 ± 0.017 | 0.000148 ± 0.000116 | 1.82e-54 ± 0.0195 | 1.02 ± 0 | 0.01 ± 0.000991 | 0.01 ± 0.000874 | 22.9 ± 2.19 | 243 ± 4.59 |
| *B. trichantha* | 86.4 ± 117 | 50 ± 446 | 40.9 ± 2.86 | 0.000774 ± 0.00105 | 0.000549 ± 0.000656 | 0.012 ± 0.0204 | 1 ± 1.59 | 0.004 ± 0.000252 | 0.00356 ± 0.000349 | 40.3 ± 3.99 | 237 ± 4.2 |
| *B. utile* | 94.6 ± 110 | 4.51 ± 144 | 37.7 ± 33 | 0.001 ± 0.0268 | 0.000384 ± 0.00383 | 0.00373 ± 0.0487 | 3 ± 24.2 | 0.00482 ± 0.000206 | 0.005 ± 0.000187 | 130 ± 20.4 | 194 ± 3.55 |
| *C. guiamemsis* | 17.2 ± 33 | 49.3 ± 7.26e+04 | 40.8 ± 5.71e+03 | 0.001 ± 0.112 | 4.81e-05 ± 0.00431 | 9.48e-53 ± 0.00756 | 1.3 ± 0 | 0.00112 ± 0.000101 | 0.00113 ± 0.000104 | 79.5 ± 18.1 | 154 ± 2.57 |
| *C. peltata* | 3.8 ± 169 | 50 ± 1.99e+04 | 42.6 ± 2.6e+03 | 0.001 ± 0.0188 | 0.00023 ± 0.00199 | 1.2e-42 ± 0.019 | 1.75 ± 0 | 0.0062 ± 0.000549 | 0.00643 ± 0.000606 | 20.4 ± 0.99 | 276 ± 3.74 |
| *C. bicolor* | 128 ± 283 | 1.81 ± 6.46 | 34.4 ± 23.3 | 0.001 ± 0.0455 | 0.0002 ± 0.000873 | 0.00189 ± 0.0297 | 3 ± 49.3 | 0.0079 ± 0.000372 | 0.0079 ± 0.000355 | 790 ± 121 | 145 ± 3.6 |
| *D. major* | 140 ± 230 | 2 ± 5.88 | 35.9 ± 8.17 | 0.000158 ± 0.000105 | 0.000778 ± 0.019 | 0.0158 ± 0.0632 | 1 ± 4.95 | 0.00617 ± 0.000243 | 0.00617 ± 0.000241 | 3.57e+03 ± 444 | 130 ± 1.29 |
| *D. major_1* | 97.9 ± 64.4 | 50 ± 173 | 37.8 ± 2.01 | 0.00088 ± 0.00138 | 0.001 ± 0.00141 | 0.0186 ± 0.0166 | 1 ± 0.551 | 0.00616 ± 0.000492 | 0.00637 ± 0.000535 | 1.73e+03 ± 394 | 134 ± 3.3 |
| *G. madruno* | 77.4 ± 51.9 | 50 ± 62.2 | 37.1 ± 0.903 | 0.000287 ± 0.000314 | 0.000227 ± 0.000156 | 8.32e-14 ± 0.0182 | 1 ± 0 | 0.00517 ± 0.000537 | 0.00518 ± 0.000149 | 893 ± 367 | 151 ± 8.52 |
| *G. dumetorum* | 39 ± 11.1 | 13.1 ± 9.55 | 34.4 ± 1.97 | 0.001 ± 0.00614 | 9.12e-05 ± 2.18e-05 | 1.52e-15 ± 0.00118 | 3 ± 0 | 0.00571 ± 0.000404 | 0.0059 ± 0.000382 | 329 ± 38.4 | 164 ± 2.4 |
| *H. scandens* | 93.9 ± 234 | 2.25 ± 40.5 | 40.8 ± 165 | 0.001 ± 0.0231 | 0.000614 ± 0.0117 | 0.0185 ± 0.408 | 1.03 ± 1.47 | 0.00387 ± 0.000147 | 0.00374 ± 0.000126 | 42.5 ± 5.89 | 195 ± 2.47 |
| *L. seemannii* | 181 ± 235 | 2.03 ± 11.2 | 38.1 ± 16.1 | 0.001 ± 0.00791 | 0.001 ± 0.00604 | 1.21e-44 ± 0.0105 | 1.97 ± 0 | 0.00884 ± 0.000321 | 0.00816 ± 0.000274 | 22 ± 2.47 | 309 ± 7.17 |
| *M. bidentata* | 200 ± 258 | 21.6 ± 3.16e+04 | 37.4 ± 346 | 0.001 ± 0.0221 | 0.000516 ± 0.0117 | 1.63e-52 ± 0.00111 | 1.26 ± 0 | 0.00141 ± 0.000207 | 0.00118 ± 0.00018 | 30.4 ± 4.29 | 193 ± 5.83 |
| *M. minutiflora* | 200 ± 194 | 1.4 ± 0.873 | 32.9 ± 2.52 | 0.000141 ± 0.00016 | 0.000277 ± 0.00107 | 2.92e-171 ± 0.0448 | 1.11 ± 0 | 0.01 ± 0.000802 | 0.01 ± 0.000561 | 47.1 ± 7.2 | 226 ± 4.74 |
| *N. cuspidata* | 18.6 ± 29.3 | 49.9 ± 1.33e+04 | 40.1 ± 805 | 0.001 ± 0.0384 | 0.000155 ± 0.00213 | 4.28e-41 ± 0.0392 | 1 ± 0 | 0.00733 ± 0.000518 | 0.00751 ± 0.000561 | 20.1 ± 1.61 | 267 ± 4.72 |
| *P. vitifolia* | 200 ± 231 | 1.82 ± 1.33 | 35.1 ± 1.75 | 0.001 ± 0.0242 | 0.000102 ± 4.55e-05 | 2.23e-14 ± 0.00986 | 1 ± 0 | 0.00221 ± 0.000203 | 0.00221 ± 0.000202 | 1e+04 ± 4.15e+03 | 129 ± 1.86 |
| *S. morototoni* | 6.88 ± 102 | 49.9 ± 4.24e+03 | 39.3 ± 294 | 0.001 ± 0.021 | 0.000195 ± 0.000794 | 4.83e-30 ± 0.0168 | 2.53 ± 0 | 0.01 ± 0.001 | 0.01 ± 0.00118 | 17 ± 1.49 | 310 ± 6.48 |
| *S. mexicana* | 122 ± 60.9 | 40.9 ± 1.34e+04 | 37.8 ± 65 | 0.001 ± 0.00397 | 0.001 ± 0.00397 | 0.0156 ± 0.0618 | 1 ± 0.807 | 0.00457 ± 0.000285 | 0.00412 ± 0.000291 | 35.7 ± 4.34 | 229 ± 4.93 |
| *S. mombin* | 92.9 ± 27.6 | 3.53 ± 2.31 | 35.1 ± 1.71 | 0.001 ± 0.00712 | 0.000142 ± 4.44e-05 | 0.000145 ± 0.00506 | 3 ± 76 | 0.00516 ± 0.00012 | 0.00509 ± 0.000261 | 23.2 ± 2.57 | 226 ± 4.84 |
| *T. pittieri* | 200 ± 220 | 1.09 ± 0.387 | 36.6 ± 6.93 | 0.000323 ± 0.00275 | 8.91e-05 ± 5.12e-05 | 1.56e-13 ± 0.0344 | 1.08 ± 0 | 0.00566 ± 0.000314 | 0.00508 ± 0.000296 | 23.9 ± 2.1 | 211 ± 3.19 |
| *V. depleta* | 126 ± 152 | 8.53 ± 321 | 36.9 ± 24.8 | 0.001 ± 0.0202 | 0.000266 ± 0.0021 | 5.14e-44 ± 0.0174 | 1.67 ± 0 | 0.00342 ± 0.000235 | 0.00342 ± 0.000235 | 1e+04 ± 2.61e+03 | 127 ± 1.26 |
| *V. multiflora* | 106 ± 259 | 22.5 ± 30 | 37.1 ± 2.7 | 0.000614 ± 0.00219 | 0.000386 ± 0.000694 | 0.013 ± 0.0168 | 1.28 ± 3.98 | 0.00195 ± 5.32e-05 | 0.00196 ± 5.2e-05 | 1.52e+03 ± 335 | 134 ± 2.55 |
| *C. fagifolia* | 106 ± 435 | 1.19 ± 3.22 | 36 ± 43.4 | 0.000159 ± 0.000611 | 0.000134 ± 0.000409 | 0.00543 ± 0.0447 | 3 ± 12 | 0.00467 ± 0.000369 | 0.00509 ± 0.00067 | measured | measured |
| *G. goudotiana* | 130 ± 238 | 2.5 ± 5.3 | 33.1 ± 3.44 | 0.000121 ± 0.000106 | 0.000734 ± 0.0593 | 0.00498 ± 0.0747 | 1 ± 14.9 | 0.00344 ± 0.000421 | 0.00394 ± 0.000424 | measured | measured |
| *I. laurina* | 95.2 ± 362 | 1.4 ± 2.98 | 37.3 ± 106 | 0.000414 ± 0.000763 | 0.00993 ± 14.6 | 0.0575 ± 0.184 | 1 ± 3.29 | 0.0017 ± 0.000182 | 0.00165 ± 0.000276 | measured | measured |
| *P. taeda n (d)* | 15 ± 4.8 | 28.1 ± 9.03 | 36.9 ± 0.736 | 7.47e-05 ± 1.35e-05 | 6.82e-05 ± 3.9e-06 | 0.00986 ± 0.00907 | 1 ± 0.426 | 0.000185 ± 7.07e-06 | 0.000185 ± 7.07e-06 | 3.48 ± 0.0529 | 313 ± 2.64 |
| *P. taeda n (ww eCO$_2$)* | 15 ± 4.8 | 28.1 ± 9.03 | 36.9 ± 0.736 | 7.47e-05 ± 1.35e-05 | 6.82e-05 ± 3.9e-06 | 0.00986 ± 0.00907 | 1 ± 0.426 | 0.00137 ± 3e-05 | 0.00136 ± 7.89e-05 | 3.2 ± 0.0149 | 343 ± 0.75 |
| *P. taeda n (ww)* | 15 ± 4.8 | 28.1 ± 9.03 | 36.9 ± 0.736 | 7.47e-05 ± 1.35e-05 | 6.82e-05 ± 3.9e-06 | 0.00986 ± 0.00907 | 1 ± 0.426 | 0.000981 ± 4.76e-05 | 0.000975 ± 4.87e-05 | 8.85 ± 0.0282 | 327 ± 0.439 |
| *P. deltoides n (d)* | 34.1 ± 2.33 | 13.2 ± 2.02 | 44.7 ± 1.22 | 0.000278 ± 5.4e-05 | 0.000327 ± 3.01e-05 | 0.00482 ± 0.00697 | 2.67 ± 1.82 | 0.000575 ± 7.87e-05 | 0.000502 ± 7e-05 | 3.51 ± 0.433 | 549 ± 45.9 |
| *P. deltoides n (ww eCO$_2$)* | 34.1 ± 2.33 | 13.2 ± 2.02 | 44.7 ± 1.22 | 0.000278 ± 5.4e-05 | 0.000327 ± 3.01e-05 | 0.00482 ± 0.00697 | 2.67 ± 1.82 | 0.0413 ± 0.00159 | 0.109 ± 0.0113 | 2.73 ± 0.157 | 231 ± 34.1 |
| *P. deltoides n (ww)* | 34.1 ± 2.33 | 13.2 ± 2.02 | 44.7 ± 1.22 | 0.000278 ± 5.4e-05 | 0.000327 ± 3.01e-05 | 0.00482 ± 0.00697 | 2.67 ± 1.82 | 0.0203 ± 0.00157 | 0.043 ± 0.00722 | 3.49 ± 0.11 | 408 ± 14.3 |

*Table S1. Fitted parameters and their standard error from the non-linear least squares fitting of photosynthesis, the leaf energy balance, and the critical stomatal conductance. Where measure values have been used in place of fitted parameters is noted.*

**Supplementary Notes S1**

To derive the modified g1 parameter we follow the methodology outlined in the supplementary material of Medlyn et al (2011) and the Appendix of Arneth et al (2002). Here we write much of their derivation for clarity, noting where our derivation diverges and highlighting where equations are comparable within our derivation and that of Medlyn et al.

Following Arneth et al., we start with the classical definition of the minimisation of the marginal water cost of plant carbon gain (Cowan 1977):

$$\begin{aligned} \frac{\partial E}{\partial A}=\lambda\#\left( S1 \right) \end{aligned}$$

where $E$ is transpiration rate, $A$is the CO_2_ assimilation rate, and $\lambda$ is a Lagrange multiplier that represents the marginal water cost of plant carbon gain.

We can also write:

$$\begin{aligned} \frac{\frac{\partial E}{\partial g_{s}}}{\frac{\partial A}{\partial g_{s}}}\boldsymbol{=}\lambda\#\left( S2 \right) \end{aligned}$$

where $g_{s}$ is the stomatal conductance.

Using simplifications, the numerator can be expressed as:

$$\begin{aligned} \frac{\partial E}{\partial g_{s}}=\frac{1}{g_{s}}E=D \#\left( S3 \right) \end{aligned}$$

where $D$ is the leaf-to-air vapor pressure difference.

And the denominator as:

$$\begin{aligned} \frac{\partial A}{\partial g_{s}}=\frac{1.6r_{s}^{2}A}{1.6r_{s}+\frac{1}{{\partial A}/{\partial C_{i}}}} \#\left( S4 \right) \end{aligned}$$

where $r_{s}$ is the stomatal resistance (equal to $1/g_{s}$), and $C_{i}$ is the intercellular partial pressure of CO_2_ within the leaf.

This is the expression used by Arneth et al., and is obtained by substituting $C_{i}$ from Fick’s law into the following expression:

$$\begin{aligned} \frac{\partial A}{\partial g_{s}}=\frac{\partial A}{\partial C_{i}}\frac{\partial C_{i}}{\partial g_{s}} \#\left( S5 \right) \end{aligned}$$

However, this does not consider the evaporative cooling effect of opening stomata. This is the point at which our derivation diverges from that of Arneth et al. as we replace equation [S5] with the following equation:

$$\begin{aligned} \frac{\partial A}{\partial g_{s}}=\frac{\partial A}{\partial T_{l}}\frac{\partial T_{l}}{\partial g_{s}}+\frac{\partial A}{\partial C_{i}}\frac{\partial C_{i}}{\partial g_{s}} \#\left( S6 \right) \end{aligned}$$

where $T_{l}$ is leaf temperature. This now takes into account the evaporative cooling effect of opening stomata.

Following through the derivation in the same way we arrive at a modified version of equation [S4]:

$$\begin{aligned} \frac{\partial A}{\partial g_{s}}=\frac{\frac{\partial A}{\partial T_{l}}\frac{\partial T_{l}}{\partial g_{s}}+1.6r_{s}^{2}A\frac{\partial A}{\partial C_{i}}}{1.6r_{s}\frac{\partial A}{\partial C_{i}}+1} \#\left( S7 \right) \end{aligned}$$

For ease here we define:

$$\begin{aligned} \xi=\frac{1}{D}\frac{\partial A}{\partial T_{l}}\frac{\partial T_{l}}{\partial g_{s}} \#\left( S8 \right) \end{aligned}$$

Now resuming the methodology in Arneth et al, we combine equations [S2], [S3], [S7] and rearranging we obtain:

$$\begin{aligned} \lambda=\frac{D\left( 1.6r_{s}+\frac{1}{{\partial A}/{\partial C_{i}}} \right)}{\frac{\xi D}{{\partial A}/{\partial C_{i}}}+1.6r_{s}^{2}A} \#\left( S9 \right) \end{aligned}$$

Note here that our equation [S9] is the modified equivalent of equation [A8] in Arneth et al. (2002).

Again following Arneth, we write:

$$\begin{aligned} A=\frac{C_{a}-C_{i}}{1.6r_{s}} \#\left( S10 \right) \end{aligned}$$

where $C_{a}$ is the partial pressure of CO_2_ in the atmosphere.

Combining equations [S9] and [S10] and rearranging we arrive at:

$$\begin{aligned} g_{s}=\frac{1.6\frac{\partial A}{\partial C_{i}}\left( \frac{\lambda\left( C_{a}-C_{i} \right)}{1.6D}-1 \right)}{1-\xi\lambda} \#\left( S11 \right) \end{aligned}$$

Again note that here our equation [S11] is the modified equivalent of equation [A11] in Arneth et al. (2002).

Again using equation [S10] we also obtain:

$$\begin{aligned} A=\frac{\frac{\partial A}{\partial C_{i}}\left\{ \lambda\left( C_{a}-C_{i} \right)^{2}-1.6D\left( C_{a}-C_{i} \right) \right\}}{1.6D\left( 1-\xi\lambda\right)} \#\left( S12 \right) \end{aligned}$$

This can be expressed as a quadratic expression with respect to $C_{i}$:

$$\begin{aligned} \alpha C_{i}^{2}+\beta C_{i}+\gamma=0 \#\left( S13 \right) \end{aligned}$$

where $\alpha=\lambda$; $\beta=1.6D-2C_{a}\lambda$; and $\gamma=\lambda C_{a}^{2}-1.6DC_{a}-\frac{1.6DA\left( 1-\xi\lambda\right)}{{\partial A}/{\partial C_{i}}}$

We need expressions for $A$ and $\frac{\partial A}{\partial C_{i}}$ and so following Arneth et al. (2002) and Medlyn et al. (2011) we use the light limited regime of the Farquhar von Caemmerer model (Farquhar et al., 1980):

$$\begin{aligned} A_{J}=\frac{J}{4}\frac{C_{i}-\Gamma^{*}}{2\Gamma^{*}+C_{i}} \#\left( S14 \right) \end{aligned}$$

where $J$ is the maximum rate of electron transport; $\Gamma^{*}$ is the CO_2_ compensation point in the light; and respiration is neglected.

Substituting [S14] and its derivative into [S13] we arrive at the following quadratic in $C_{i}$:

$$\begin{aligned} aC_{i}^{2}+bC_{i}+c=0 \#\left( S15 \right) \end{aligned}$$

where:

$$a=3\Gamma^{*}-L\left( 1-\xi\lambda\right)$$

$$b=2\Gamma^{*}\left( L-3C_{a} \right)+\xi\lambda L\Gamma^{*}$$

$$\begin{aligned} c=L\left( 2{\Gamma^{*}}^{2}\left( 1-\xi\lambda\right)-3C_{a}\Gamma^{*} \right)+3C_{a}^{2}\Gamma^{*} \#\left( S16 \right) \end{aligned}$$

where $L$ represents the combination of terms ($=1.6D/\lambda$)

Note this is now the related expression to equation [A1] of Medlyn et al. (2011). It is useful to note here that when $\xi=0$ (i.e. when there is no evaporative cooling), the equations become exactly equivalent.

Following Medlyn et al., we calculate the discriminant ($\Delta=b^{2}-4ac$) of this quadratic:

$$\begin{aligned} \Delta=12\Gamma^{*}L\left[ \left( 1-\xi\lambda\right)\left( C_{a}^{2}+\Gamma^{*}C_{a}-2\Gamma^{*^{2}} \right)-L\left( C_{a}-\Gamma^{*} \right)-\frac{D\xi\lambda\left( 4-\xi\lambda\right)}{12}L\Gamma^{*} \right] \#\left( S17 \right) \end{aligned}$$

If we assume that $C_{a}\gg\Gamma^{*}$, this expression simplifies to

$$\begin{aligned} \Delta\approx12L\Gamma^{*}\left( 1-\xi\lambda\right)C_{a}^{2} \#\left( S18 \right) \end{aligned}$$

The remainder of the derivation follows Medlyn et al., with our modified quadratic and discriminant. We calculate a $C_{i}:C_{a}$ ratio as:

$$\begin{aligned} \frac{C_{i}}{C_{a}}\approx\frac{3\Gamma^{*}-\sqrt{3L\Gamma^{*}\left( 1-\xi\lambda\right)}}{3\Gamma^{*}-L\left( 1-\xi\lambda\right)} \#\left( S19 \right) \end{aligned}$$

Which can be simplified by completing the square in the denominator, giving:

$$\begin{aligned} \frac{C_{i}}{C_{a}}\approx\frac{\sqrt{3\Gamma^{*}}}{\sqrt{3\Gamma^{*}}+\sqrt{L\left( 1-\xi\lambda\right)}} \#\left( S20 \right) \end{aligned}$$

Rearranging equation [S10] and substituting equation [S20] we arrive at:

$$\begin{aligned} g_{s}\approx1.6\left( 1+\sqrt{\frac{3\Gamma^{*}\lambda}{1.6D\left( 1-\xi\lambda\right)}} \right)\frac{A}{C_{a}} \#\left( S21 \right) \end{aligned}$$

Re-writing this equation in the form of the classic Medlyn model we arrive at the main text expression of:

$$\begin{aligned} g_{s}\approx1.6\left( 1+\frac{g_{1}^{*}}{\sqrt{D}} \right)\frac{A}{C_{a}} \#\left( S22 \right) \end{aligned}$$

Where the parameter $g_{1}^{*}$ is the modified $g_{1}$ parameter given by:

$$\begin{aligned} g_{1}^{*}=\sqrt{\frac{3\Gamma^{*}\lambda}{1.6\left( 1-\xi\lambda\right)}} \#\left( S23 \right) \end{aligned}$$

**References**

Arneth, A., Lloyd, J., ˇSantr˚uˇckov´a, H., Bird, M., Grigoryev, S., Kalaschnikov, Y. N., Gleixner, G., and Schulze, E.-D. (2002). Response of central siberian scots pine to soil water deficit and long-term trends in atmospheric co2 concentration. Global Biogeochemical Cycles, 16(1):5–1–5–13. doi:[10.1029/2000GB001374](https://doi.org/10.1029/2000GB001374)

Cowan, I. R. and Farquhar, G. D. (1977). Stomatal function in relation to leaf metabolism and environment. Symp Soc Exp Biol, 31:471–505.

Diao, H., Cernusak, L.A., Saurer, M., Gessler, A., Siegwolf, R.T.W. and Lehmann, M.M. (2024), Uncoupling of stomatal conductance and photosynthesis at high temperatures: mechanistic insights from online stable isotope techniques. New Phytol, 241: 2366-2378. <https://doi.org/10.1111/nph.19558>

Farquhar, G. D., von Caemmerer, S., and Berry, J. A. (1980). A biochemical model of photosynthetic co2 assimilation in leaves of c3 species. Planta, 149(1):78–90.

Medlyn, B. E., Duursma, R. A., Eamus, D., Ellsworth, D. S., Prentice, I. C., Barton, C. V. M., Crous, K. Y., De Angelis, P., Freeman, M., and Wingate, L. (2011). Reconciling the optimal and empirical approaches to modelling stomatal conductance. Global Change Biology, 17(6):2134–2144. <https://doi.org/10.1111/j.1365-2486.2010.02375.x>
